# Unrolled-DOT: an interpretable deep network for diffuse optical tomography

**DOI:** 10.1117/1.JBO.28.3.036002

**Published:** 2023-03-08

**Authors:** Yongyi Zhao, Ankit Raghuram, Fay Wang, Stephen Hyunkeol Kim, Andreas Hielscher, Jacob T. Robinson, Ashok Veeraraghavan

**Affiliations:** a Rice University, Department of Electrical and Computer Engineering, Houston, Texas, United States; b Columbia University, Department of Biomedical Engineering, New York, New York, United States; c Columbia University Irvine Medical Center, Department of Radiology, New York, New York, United States; d New York University - Tandon School of Engineering, Department of Biomedical Engineering, New York, New York, United States

**Keywords:** optical tomography, machine learning, time-of-flight imaging

## Abstract

**Significance:**

Imaging through scattering media is critical in many biomedical imaging applications, such as breast tumor detection and functional neuroimaging. Time-of-flight diffuse optical tomography (ToF-DOT) is one of the most promising methods for high-resolution imaging through scattering media. ToF-DOT and many traditional DOT methods require an image reconstruction algorithm. Unfortunately, this algorithm often requires long computational runtimes and may produce lower quality reconstructions in the presence of model mismatch or improper hyperparameter tuning.

**Aim:**

We used a data-driven unrolled network as our ToF-DOT inverse solver. The unrolled network is faster than traditional inverse solvers and achieves higher reconstruction quality by accounting for model mismatch.

**Approach:**

Our model “Unrolled-DOT” uses the learned iterative shrinkage thresholding algorithm. In addition, we incorporate a refinement U-Net and Visual Geometry Group (VGG) perceptual loss to further increase the reconstruction quality. We trained and tested our model on simulated and real-world data and benchmarked against physics-based and learning-based inverse solvers.

**Results:**

In experiments on real-world data, Unrolled-DOT outperformed learning-based algorithms and achieved over 10× reduction in runtime and mean-squared error, compared to traditional physics-based solvers.

**Conclusion:**

We demonstrated a learning-based ToF-DOT inverse solver that achieves state-of-the-art performance in speed and reconstruction quality, which can aid in future applications for noninvasive biomedical imaging.

## 1 Introduction

Light scattering is one of the primary limitations for optical imaging in biological tissue. It leads to lower spatial resolution, contrast and signal-to-noise ratio (SNR). Many optical imaging methods attempt to mitigate the effects of scattering through ballistic imaging, filtering unscattered photons from heavily scattered photons. A number of ballistic imaging techniques have been developed, including spatial filtering (i.e., confocal microscopy), polarimetry, and time-of-flight (ToF) imaging.^1^^–^^4^ Unfortunately, the number of ballistic photons decreases exponentially with optical thickness. For example, optical imaging through the human skull requires imaging through ∼6.5  mm of tissue with scattering coefficient $9\;{\mathrm{mm}}^{-1}$. This corresponds to ∼60 mean free paths, which indicates on average a photon will undergo 60 scattering events while propagating through a scatterer of this thickness. Therefore, only one ballistic photon would remain for every $e^{60}=1.14*10^{26}$ photons from the light source. As a result, most ballistic imaging methods fail to image beyond a few millimeters of tissue. The limited depth of ballistic imaging has led to great interest in diffuse optical tomography, a technique that uses ballistic and diffuse photons to image through densely scattering media.

In diffuse optical tomography (DOT), an array of light sources and detectors, which are placed on the subject, capture a set of optical measurements. An associated reconstruction algorithm converts these measurements into a high resolution, human-interpretable images. To establish this reconstruction algorithm, DOT uses the predictable relationship between spatial variations in the optical properties and the spatial-angular distribution of light, the radiance.^5^^,^^6^ This relationship can be represented as a function known as the forward model, which maps the spatial distribution of optical properties to the radiance. To perform image reconstruction, this forward model must be inverted, establishing a mapping from the radiance to the spatial distribution of optical properties. This is generally referred to as the inverse problem. It is widely accepted that an accurate forward model and inverse solver are essential to high accuracy DOT image reconstruction.^7^ Accurate forward models and inverse solvers were essential in several recent papers that achieved high resolution reconstructions through densely scattering media.^5^^,^^8^^–^^11^

Despite the recent progress in optimizing the DOT inverse solver, it still possesses several limitations. First, inverse solvers can be slow. Non-linear algorithms may require several hours to complete.^12^ Even linear inverse algorithms, which are regarded as the fastest solvers, may require minutes to reconstruct a single image.^9^ This precludes the possibility of real-time imaging of biological events, which may require subsecond timing resolution. Second, many inverse solvers require several hyperparameters, which are hand-tuned. Several prior works use optimization-based inverse solvers that include regularization terms, such as Tikhonov or $L^{1}$ norm regularization. Scaling the weights of these regularization terms can impact the resulting image reconstruction. For example, increasing $L^{1}$ norm regularization increases image sparsity. This can improve the image reconstruction quality. However, since the parameters are hand-tuned, there is no guarantee that the final result achieves the highest fidelity to the original image. Third, the image reconstruction quality is highly sensitive to the system calibration. Reconstruction algorithms require knowledge of the system parameters, i.e., the source and detector positions, the target geometry, and the camera transform. While these parameters can sometimes be estimated through a calibration procedure, this can be difficult for dynamic or living targets. This can lead to model mismatch in estimating the background measurement^13^ or the mesh geometry^14^ and can lead to inaccurate reconstructions of the target’s optical properties or position.

**Fig. 1:**
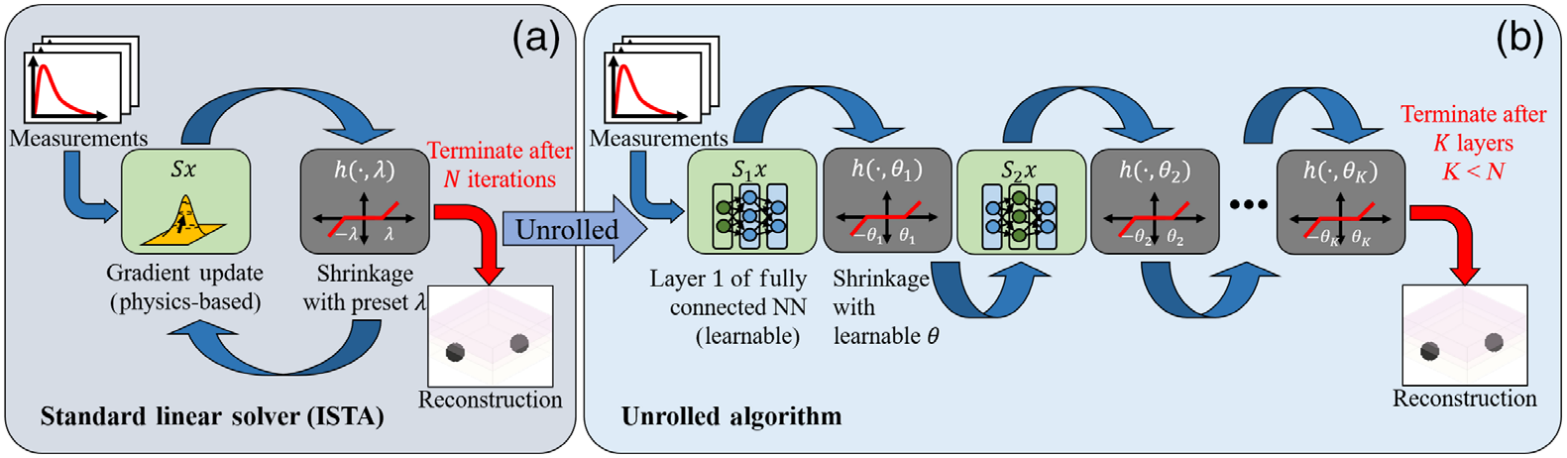
Comparison of unrolled algorithms versus standard iterative linear solvers: (a) classical inverse solvers, such as ISTA perform reconstruction by iteratively applying a gradient step followed by the proximal operator, i.e., the shrinkage function with predetermined parameter λ. (b) This iterative procedure can be unrolled. The gradient steps become FC layers of a neural network. The non-differentiable operation becomes an activation function, such as a shrinkage function with learnable parameter θ. Because the forward model is learned, unrolled algorithms can reduce the number of layers and perform faster reconstruction compared to linear solvers. The learned model can also reduce model mismatch and increase the image reconstruction quality.

To address these challenges, we propose to use algorithm unrolling as the DOT inverse solver, a technique we have dubbed “Unrolled-DOT.” In algorithm unrolling, the iterations of a standard linear inverse solver are converted into fully connected (FC) layers of a neural network.^15^^,^^16^ This has been shown to improve the image reconstruction quality and runtime of the solver (Fig. 1). As shown by Lecun et al.,^16^ and as we will demonstrate in Sec. 6, the unrolled algorithm can achieve a significantly faster algorithm runtime than traditional physics-based solvers. In addition to improving the runtime, Unrolled-DOT also improves on traditional physics-based solvers since it directly learns the hyperparameters and calibration from data. In addition to outperforming physics-based solvers, there are also benefits to using the unrolled algorithm versus other learning-based models: the unrolled algorithm is interpretable, and can be trained with fewer parameters than traditional networks.^15^ While many traditional deep learning models are considered “black-box models,” the learned parameters of the unrolled-algorithm correspond to physically meaningful values. For example, the learned activation parameter in the unrolled network corresponds to sparsity regularization. In addition, traditional methods may possess tens of millions of parameters and require millions of examples for training.^17^^,^^18^ We will demonstrate that Unrolled-DOT can be trained with just a few thousand examples.

Thus, we report the following contributions.

1. Our algorithm achieves higher image reconstruction quality compared to both physics-based and learning-based solvers for DOT/ToF-DOT image reconstructions. Our method reduces the mean-squared error (MSE) by over an order of magnitude on real-world data, compared with physics-based linear solvers.
2. Our method is faster than traditional linear DOT solvers, achieving over an order of magnitude reduction in the runtime compared to all other physics-based methods in our comparison. High-quality image reconstruction can be achieved with just one layer, approximately corresponding to a single iteration of a standard linear solver. In contrast, other linear inverse solvers may require tens of iterations.
3. We collected the first real-world DOT/ToF-DOT dataset with thousands of measurement and ground truth pairs (5000 total), which we made available to the public.^19^ In generating DOT tissue phantoms, it is difficult to precisely place the absorbing targets and even harder to achieve on a large scale. Our method uses an Eink display to programmatically control the target shape and placement, which provides a more accurate ground truth. This is important for supervised learning algorithms that require measurement and ground-truth pairs. In addition, these supervised models typically require thousands of training examples for DOT reconstruction,^20^^–^^22^ which could only be achieved with synthetic datasets in prior works. In the future, our real-world dataset could be used to train and benchmark models on data that inherently possesses the features (noise, contrast, etc.) of real-world measurements. This can lead to more accurate benchmarks and trained algorithms that better account for model mismatch with real-world measurements.

While our approach makes several improvements over existing methods, we also acknowledge that there are certain limitations that can be addressed in future work. First, our prototype uses a single laser source and single-pixel detector, which are raster-scanned by galvo mirrors. In the future, an imaging system that possesses an on-chip array of sources and detectors would improve the measurement acquisition speed. In addition, the measurements are currently captured for only two-dimensional imaging targets and can be extended to three-dimensional targets. In future work, it may be possible to extend to other imaging modalities through transfer learning or by learning only the parameters associated with the physical apparatus. These learned parameters can be treated as a calibration. To perform image reconstruction, the physics-based models can still be applied on top of this calibration. Finally, applying these algorithms to *in-vivo* targets is reserved as future work.

## 2 Related Works

### 2.1 DOT/ToF-DOT

There is a long history of imaging through scattering media using techniques such as near-infrared spectroscopy (NIRS) and diffuse optical tomography (DOT). Early works in NIR spectroscopy include monitoring of blood oxygenation levels in the brain, which began in the 1970s.^23^ It was later discovered that neural activity was correlated to blood oxygenation levels, and could be measured using NIRS, which led to the development of functional near infrared spectroscopy in the 1990’s.^24^ In the present day, DOT has been applied to many applications in biomedical imaging, including functional neuroimaging, breast tumor detection, and small animal imaging.^25^^–^^27^ DOT techniques can be generally categorized into three modalities: continuous-wave (CW), frequency domain (FD), and time domain (TD), which is also referred to as ToF.

In CW-DOT, each recorded measurement between a source and detector is a scalar intensity value. Due to the simplicity of the associated hardware, this technology is the most cost-efficient and can be easily integrated into wearable devices.^28^ CW-DOT has been shown to image up to centimeters deep into tissue, but generally achieves poorer spatial resolution on the order of centimeters.^6^ Recent progress with high-density DOT, which uses a dense array of sources and detectors, has shown improvement in spatial resolution.^25^ Another DOT imaging modality is FD diffuse optical tomography (FD-DOT). FD-DOT uses a frequency-modulated optical signal for the light source. The optical properties of the target change the amplitude and phase of the resulting measurements, which can improve the image reconstruction quality using greater measurement diversity.^29^

Finally, the third DOT imaging modality is TD or ToF diffuse optical tomography (ToF-DOT), which enhances the imaging quality using TD measurements on the order of picoseconds. While the theory for ToF-DOT has existed since the 1990s, development of the technology was limited by hardware performance and cost.^30^ In recent years, ToF-DOT imaging systems have risen in popularity as the technology for TD detectors has matured. Lyons et al.^9^ demonstrated that a transmission-geometry ToF-DOT system could achieve approximately millimeter-scale spatial resolution through a 2.5-cm-thick scatterer. Unfortunately, ToF-DOT systems were still very slow due to the large volume of spatial-temporal measurements that had to be processed. Thus, confocal scanning geometries (CToF-DOT) have been applied to ToF-DOT, reducing the number of measurements that are required while maintaining a high spatial resolution.^5^^,^^10^

### 2.2 Physics-based DOT Inverse Solvers

While many imaging modalities exist, all DOT techniques require a forward model of light propagation as well as an inverse solver to recover a spatial distribution of optical properties from optical measurements. Both the forward model and inverse solver have an enormous impact on the image reconstruction quality and the overall runtime. Most algorithms exhibit a tradeoff between the reconstruction quality and runtime. Therefore, the forward and inverse algorithm selection is critical. The most accurate method of light propagation is generally a full solution to the radiative transfer equation using the Monte Carlo algorithm.^6^^,^^31^^–^^33^ While this approach is accurate, it can also be quite slow. Solutions can also often be estimated using mesh-based methods, such as finite-element method (FEM) or boundary element method,^34^^–^^36^ and finite volume methods (FVM).^12^ Faster but more inaccurate forward models using the diffusion approximation^37^^,^^38^ or truncated spherical harmonics approximations^39^^–^^41^ have also been proposed. While physics-based methods have allowed DOT to achieve high resolution imaging through densely scattering media, we believe they still possess certain limitations, including a tradeoff between speed and reconstruction quality, a need for hyperparameter tuning,^5^ and a need for system calibration.^13^^,^^14^ We believe that a learning-based method may help to mitigate these challenges.

### 2.3 Data-driven Models

While a lot of efforts have been placed on physics-based approaches, there has been a rise in the popularity of machine-learning methods. Table 1 summarizes these recent works. Yedder et al. was one of the first to use a deep neural network as the DOT inverse solver.^20^ Subsequent work by Sabir et al.^21^ and Yoo et al.^22^ also applied neural networks, more specifically convolutional neural network architectures, for solving the DOT inverse problem. Nizam et al. and Deng et al. have demonstrated progress in improving CW diffuse optical tomography reconstruction quality using a variation of the automated transform by manifold approximation (AUTOMAP) architecture.^43^^–^^45^ Li et al.^46^ has applied deep learning and DOT to clinical applications, improving the accuracy of breast tumor imaging. Deep learning has also been applied for generating data in DOT applications.^47^ Recently, Zou et al.^42^ used an autoencoder to learn the forward model and inverse problem of FD-DOT. While their training data included experimental data, it was limited to only 30 unique configurations. Our proposed approach aims to improve on prior work by constructing a neural network that is more interpretable and is trained on a real-world dataset with thousands of examples to account for model mismatch.

**Table 1:** Comparison of DOT dataset size in recent works: Above, we summarize the important attributes of datasets from recent works on machine learning and DOT. For the dataset size, we break down whether the dataset was obtained through simulated or real-world data. In addition, we note the size of the data for training and testing. Most data-driven DOT algorithms are trained on large simulated datasets or a limited number of real-world measurements. Our method is the first to use a dataset of thousands of real-world measurements.

Dataset	DOT modality (CW, FD, ToF)	Real-world training data? (Y/N)	Simulated dataset size (number of Measurements)	Real dataset size (number of measurements)
Yedder et al.^20^	CW	N	4500	32 (testing only)
Sabir et al.^21^	FD	N	6500	2 (testing only)
Yoo et al.^22^	FD	N	1500	3 (testing only)
Zou et al.^42^	FD	Y	NA (trained on real-world data)	114 (24 inhomogeneous/ 90 homogeneous phantoms)
Unrolled (ours)	CW/ToF	Y	NA (trained on real-world data)	**5000** **(training** **and testing)**

The techniques described above all applied machine learning to improve the inverse solver for recovering spatial distributions of optical properties. There are also many other works that apply machine learning to other modalities of optical imaging,^48^^,^^49^ for example fluorescence molecular tomography,^50^^,^^51^ fluorescence lifetime imaging,^52^^–^^54^ and direct optical imaging without an inverse solver.^55^ In addition, machine learning has also been used in hybrid imaging modalities. For example, deep learning has improved the reconstruction quality of magnetic resonance imaging (MRI)-guided DOT/NIRS in the applications of breast tumor detection^56^ and cerebral subdural hematoma monitoring;^57^ however, because these methods also rely on MRI, they may lack the cost-efficiency and portability of an exclusively optical imaging approach.

### 2.4 Algorithm Unrolling

Neural networks are commonly treated as black boxes, hindering interpretability. This lack of explainability has limited their adoption in several domains, especially for medical imaging, in which the interpretability of results is essential. To increase the explainability of these networks, a new technique known as algorithm unrolling has risen in popularity.^15^ Gregor and Lecun were the first to propose this type of neural network architecture, an algorithm that they coined “learned iterative shrinkage thresholding algorithm” (LISTA).^16^ The key idea of this approach is to represent the iterations of the ISTA as layers of a neural network. This model is more interpretable than a standard neural network because the learnable parameters represent the forward matrix of the underlying physical model. This work has led to the “unrolling” of other algorithms, such as the alternating direction method of multipliers (ADMMs)-CSNET algorithm, and has been used in several imaging applications, such as lensless imaging and MRI.^58^^,^^59^ Recent works have also tested unrolled networks that do not train the network weights. Liu et al.^60^ introduced a network with analytically determined weights and learnable stepsizes and thresholds. In the application of diffuse optical tomography, Hua et al.^61^ used an unrolled network for fluorescence diffuse optical tomography. However, their method learns only the regularizer term rather than the entire forward model, and was trained only on simulated data, which may lead to model mismatch.

## 3 Physics-Based DOT Forward Model

The key to image reconstruction in DOT is an accurate model of light propagation, generally referred to as the forward model. The forward model establishes the relationship between the spatial distribution of optical parameters, i.e., the target image that we ultimately want to solve for, and the set of recorded measurements. Because of this relationship, the accuracy of the forward model is essential to obtaining high-quality, accurate image reconstructions. For example, if the forward model assumes an inaccurate position for the detector, this can lead to misplacement of the targets or other artifacts in the reconstructed image.

To establish an accurate forward model, we employ the radiative transfer equation (RTE). The RTE, and consequently most DOT forward models, uses the particle model of light because the wave nature of light has negligible impact on heavily scattered light. While the RTE is generally considered the most accurate model of light propagation,^39^ it can be computationally expensive or even intractable to solve for an arbitrary target geometry and imaging setup.

We model the propagation of light using a linear forward model. This model assumes a linear relationship between small changes in the optical parameters (perturbations) and the optical measurements that we collect. This is valid under the Born approximation for small optical changes, relative to the background optical properties.^31^ The linear forward model can be expressed as

Δm=JΔμ. (1)

Here, Δm, Δμ, and J represent the differential measurements, the target, and the Jacobian (or sensitivity) matrix, respectively. The target Δμ is modeled as an optical perturbation. For example, neural activity can lead to optical changes in the associated regions of the brain due to the hemodynamic response.^25^ The differential measurements $\Delta m=m_{\mathrm{bkg}}-m_{\text{target}}$ correspond to the difference between the detected intensity in the unperturbed ($m_{bkg}$) and perturbed ($m_{\text{target}}$) state. The Jacobian matrix relates these two quantities, i.e., a change in the optical parameters (Δμ) corresponds to a change in the detected intensity (Δm). Thus, each entry of the Jacobian matrix is a partial derivative: $J_{rs}=\frac{\partial m_{r}}{\partial \mu_{s}}$.

Under this linear setup, the Jacobian matrix is essential to both generating synthetic test data and image reconstruction with a traditional, physics-based forward model. Several methods exist for simulating the Jacobian matrix, including analytical expressions, FEM, and the Monte Carlo (MC) algorithm.^31^^,^^37^^,^^62^^,^^63^ We use MC in this work since it is regarded as the most accurate and flexible of these methods. The details of the implementation are described in Sec. 5; here, we will provide a brief description of the algorithm itself. MC uses randomly sampled photon trajectories to simulate the propagation of light.^64^ These photon trajectories are sampled from a distribution that incorporates the optical parameters of the target medium. By averaging the information over many samples, we obtain an accurate estimate of the distribution of light. MC is an unbiased estimator and can be easily adapted to arbitrary geometries; however, it may require many random samples and consequently a long run time to converge to a sufficiently low-variance estimate.^32^

While the RTE and MC can be used to model the underlying physics of the imaging system, they require knowledge of the imaging setup and target parameters. These parameters are provided as input to the algorithm. Some examples of input parameters include the source–detector positions which are generally a vector of coordinates and the target geometry which can be represented as a mesh. Certain parameters may be simple to estimate for a particular setup, such as the source–detector separation.^65^ Other parameters may be challenging to estimate through calibration, which can lead to model mismatch. For example, Reisman et al. showed that assuming an incorrect target geometry led to an incorrect depth estimation.^14^ To avoid potential reconstruction errors from inaccurate forward model parameters, we use the unrolled network to learn the system parameters. Thus, our training procedure can be interpreted as a calibration step. By training on real-world measurements and their associated ground truth images, our model reduces the potential for model mismatch and improves the image reconstruction quality.

## 4 Unrolled-DOT

### 4.1 Unrolled Network Design

Our ultimate goal is to solve the inverse problem: determine the target parameters from a given set of measurements. Using the linear forward model described in Eq. (1), the inverse can be formulated as a convex optimization problem

$$\Delta \mu^{*}=\underset{\Delta \mu }{\min}{\left\|J\Delta \mu -\Delta m\right\|}_{2}+\Lambda (\Delta \mu ).$$ (2)

Here, as in Sec. 3, the terms Δμ, J, Δm represent the target, Jacobian matrix, and observed differential measurements, respectively. ${\left(\cdot \right)}^{*}$ denotes the optimum. This equation states that the desired reconstruction ($\Delta \mu^{*}$) minimizes the MSE between the predicted and the observed measurements (${\left\|J\Delta \mu -\Delta m\right\|}_{2}$) and includes a penalty term (Λ(Δμ)) that enforces prior information regarding the target. For example, $\Lambda (\Delta \mu )=\lambda_{1}{\left|\Delta \mu \right|}_{1}$ would enforce an image sparsity prior. A suite of techniques exist for solving linear inverse problems that follow the form of Eq. (2). This includes the ISTA, fast ISTA (FISTA) an accelerated version of ISTA, and the ADMM.^66^^,^^67^ We will focus on ISTA because this algorithm forms the foundation of our proposed method. However, our results will include comparisons with these other standard solvers.

ISTA can be considered as a variation of gradient descent. As stated before, the problem setup generally follows a similar structure to Eq. (2), where Δμ in our notation is the standard unknown variable, and J is the standard forward matrix (often referred to as A). Using ISTA, the solution is obtained by iteratively performing the gradient step and applying a proximal operator for the non-differentiable portion of the objective. Mathematically, a single step of this procedure can be expressed as^66^

$$\Delta \mu_{k+1}=h(\Delta \mu_{k}-\gamma (J^{T}J\Delta \mu_{k}-J^{T}\Delta m);\lambda ),$$ (3)

where, $\gamma (J^{T}J\Delta \mu_{k}-J^{T}\Delta m)$ is the gradient step with step-size γ, and h(·,λ) is the proximal operator. Generally, the gradient step solves the linear inverse problem, while the proximal operator is used to enforce regularization constraints. For example, an $L^{1}$ norm penalty can be used to enforce image sparsity in the reconstruction. In this case, the proximal operator is the shrinkage function with tuning parameter λ. An overview of this procedure is also shown in Fig. 1(a). While this procedure is simple and sufficiently general that it can account for even non-differentiable terms in the objective function, there are also several challenges to using this approach, particularly for DOT image reconstruction.

First, the ISTA algorithm possesses relatively slow convergence. Even for algorithms such as FISTA, which accelerates the convergence of ISTA, tens of iterations may be needed to converge to a sufficiently accurate reconstruction. For this reason, unrolled algorithms have been proposed with the potential to increase the reconstruction speed. This gain was demonstrated by Gregor et al.^16^ and is shown empirically in our results in Sec. 6. In addition to improvements in efficiency, there are also potential benefits to the image reconstruction quality. Any misalignment between the expected forward model and the underlying system that generates the measurements can reduce the efficacy of standard linear solvers. To address this, we propose a deep network to learn the parameters of the standard inverse solver. More specifically, we apply the learned-ISTA algorithm.^16^

The learned algorithm follows a very similar structure to ISTA. Each “layer” of the algorithm corresponds to an iteration of ISTA. The i’th layer can be represented mathematically as the function^16^

$$\Delta \mu_{i+1}=f_{i}(\Delta \mu_{i})=h(S\Delta \mu_{i}+W\Delta m;\lambda ).$$ (4)

Here, W, S, and λ correspond to learnable parameters of the network. h(·;λ) is the non-linearity applied after each layer of the network and corresponds to the shrinkage function used in ISTA. Using this function, we can express the full network with L-layers as

$$F_{L}=f_{L}\circ f_{L-1}\circ ,\ldots ,\circ f_{0}.$$ (5)

**Fig. 2:**
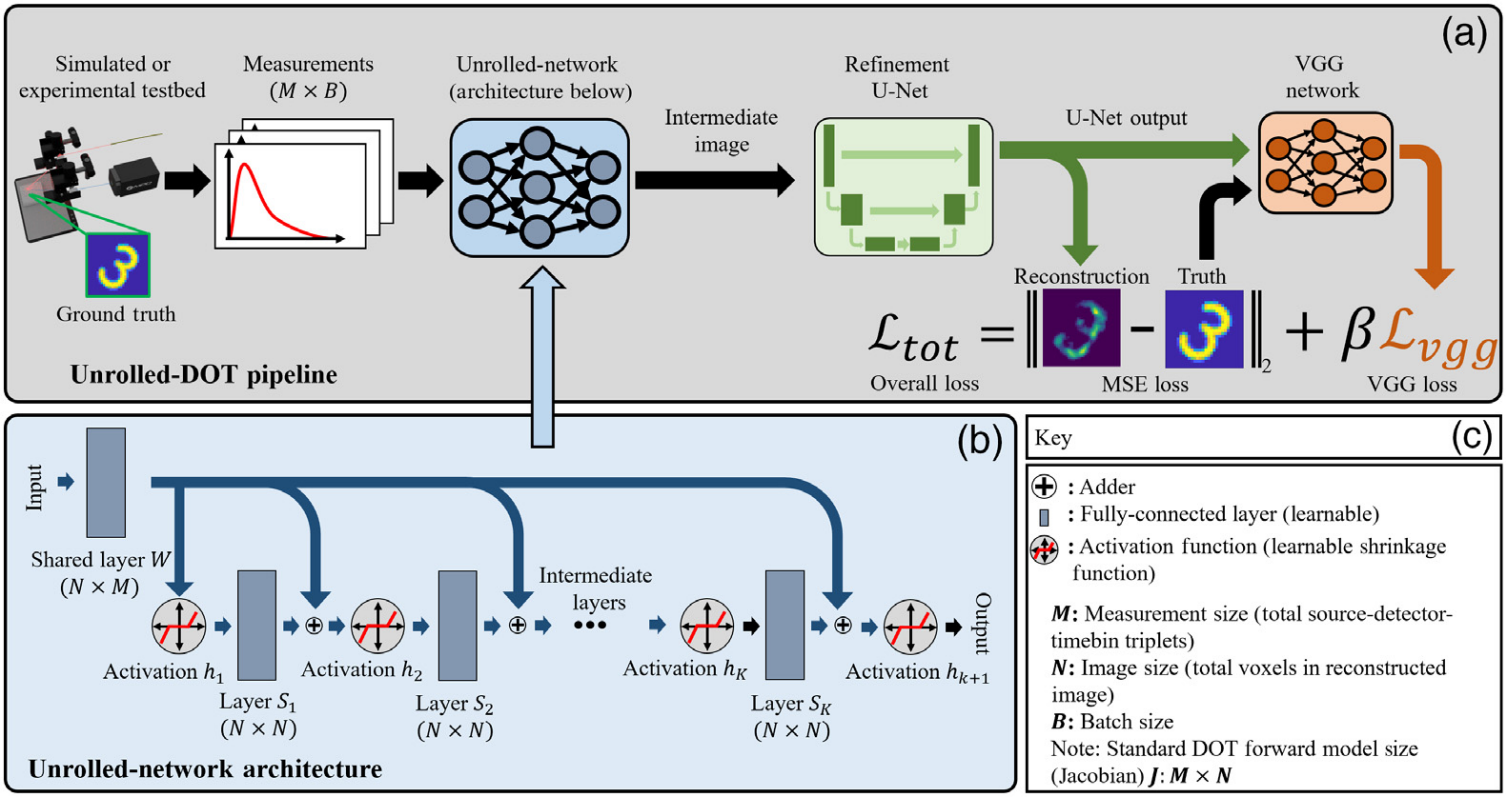
Unrolled-DOT network architecture: (a) the full algorithm pipeline is shown in the grey box. The set of measurements is first passed through an unrolled network to perform image reconstruction. Additional details are shown in panel (b); a key of relevant terms is shown in panel (c). The unrolled network uses a shared FC layer and K independent FC layers. Additionally, we use the shrinkage function as the non-linear activation function. The shrinkage functions are applied after each layer. After the unrolled network, the output is passed through a U-Net, which further improves the image reconstruction quality. The loss is a weighted sum of a standard loss (MSE or MAE) and the Visual Geometry Group (VGG) perceptual loss with weight parameter β. Backpropagation is then applied to train the unrolled network and U-Net.

Here f is the function: $f_{i}(y)=h(Sf_{i-1}(y)+Wy;\lambda )$, and $f_{0}(y)=h(Wy)$ is the “0-th” layer. An overview of this full pipeline is shown in Fig. 2(b). Next, we can show the relationship between the ISTA and the unrolled network. If we set $W=\gamma J^{T}$ and $S=I-\gamma J^{T}J$, we return to the original ISTA expression. In this case, the layers of the unrolled-network would be equivalent to the iterations of ISTA, as represented in Eq. (3) and stated in Gregor et al.^16^ One of the benefits of this type of “unrolling” algorithm is interpretability: the learned parameters provide insight into the physics of the underlying system because the layers correspond to iterations of a standard linear solver. Additional details about this are provided in Sec. 6.

Training is performed using the standard backpropagation approach, with either synthetic or experimentally collected data

$$W^{*},S^{*},\lambda^{*}=\underset{W,S,\lambda }{\min}{\left\|F(\Delta m;W,S,\lambda )-\Delta \mu \right\|}_{2}.$$ (6)

As before, W, S, and λ are parameters of the learned network, F. Here, we use MSE as the loss function to determine optimality, though other metrics can also be chosen. The measurements Δm are generated either synthetically by applying the physics-based forward model or captured experimentally using a prototype system. For training, ground truth data is accessible both in simulation and in experiments. In simulation, the ground truth and forward model directly compute the measurements. In experiments, we display the ground truth on an electronic ink (E-ink) display, which can programmatically control the spatial distribution of optical absorbers and scatterers. We collect measurements corresponding to the ground truth target with the experimental prototype. Additional details about our full training and testing procedure are described in Sec. 5.

### 4.2 Additional Perceptual Enhancement

While the unrolled network offers a powerful learning-based model for solving linear inverse problems, the quality of our image reconstructions can still be further improved. To this end, we also incorporated a refinement U-Net and VGG perceptual loss in our network design and training procedure. In adding these steps, we were inspired by Khan et al.,^68^ who demonstrated that the U-Net and VGG-loss could enhance image reconstruction quality in the application of lensless imaging.

The U-Net architecture is a convolutional neural network that uses a contracting and expanding path. This network architecture was originally designed by Ronneberger et al. for the application of image segmentation.^69^ It was subsequently shown that the U-Net architecture could also be applied to image reconstruction.^70^ In addition to the refinement U-Net, we also apply a VGG perceptual loss to further enhance the image reconstruction quality.^71^^,^^72^ The resulting loss term can be expressed as

$$W^{*},S^{*},\lambda^{*},\theta^{*}=\underset{W,S,\lambda ,\theta }{\min}{\left\|U_{\theta }(F(\Delta m;W,S,\lambda ))-\Delta \mu \right\|}_{2}+{\left\|f_{\mathrm{VGG}}(U_{\theta }(F(\Delta m;W,S,\lambda )))-f_{\mathrm{VGG}}(\Delta \mu )\right\|}_{2}.$$ (7)

For the VGG-loss, the ground truth image and the reconstructed image, i.e., the output of the refinement U-Net, are passed through a pretrained VGG network. The VGG loss is then calculated from the standard (MSE or MAE) loss between intermediate outputs of the VGG network for the predicted and true images. The overall loss then becomes the weighted sum of the standard loss and the VGG loss, as shown in Eq. (7). Here, $U_{\theta }(\cdot )$ refers to the U-Net with trainable parameters θ, and $f_{\mathrm{VGG}}(\cdot )$ is a function that maps the input to the intermediary outputs of the VGG network. Because the VGG network is pretrained, its parameters are not updated during the Unrolled-DOT training. Finally, this overall loss is backpropagated to train the unrolled network and U-Net.

## 5 Materials and Methods

### 5.1 Computational Processing

The computational processing in these experiments includes the generation of synthetic data, machine learning for training and testing our models, and computational post-processing, which includes testing other standard linear solvers. The model training and testing were implemented in Python using the Pytorch library. In addition, the structural similarity (SSIM) loss in Sec. 6.3 used the Kornia library.^73^ MATLAB was generally used for post-processing, such as plotting and visualization, as well as implementing the FISTA, and ADMM solvers for the algorithm comparison in Sec. 6.4.

The synthetic data was generated using an in-house Monte Carlo simulator, which implemented the Monte Carlo multi-layered (MCML) algorithm.^64^ The standard MNIST and Fashion MNIST images were scaled to 41×41  pixels, where each pixel corresponds to 1 mm in world coordinates. The pixel values of the images were converted to absorption coefficients, with the maximum pixel intensity scaling to $\mu_{a}=1.0\;{\mathrm{mm}}^{-1}$, and negligible background absorption. In addition, the background scattering coefficient was set to $\mu_{s}=9.0\;{\mathrm{mm}}^{-1}$. For the simulated experiments testing the generalizability of the model and the comparison of reconstruction with and without the U-Net and VGG loss a 10×10 array of sources and detectors is used. For each source–detector pair, a TD measurement consisting of four timebins is simulated. Measurements are normalized to 1.0. For the circular phantoms experiments in the supplementary material, a circular grid of 32 source–detector pairs are distributed uniformly over the circle. Again, each time-domain measurement consists of 4 timebins. We used a precomputed Jacobian matrix to apply the forward model on the ground truth and generate the full synthetic dataset. Jacobian matrix construction is described in reference.^31^ In the experimental reconstruction and simulated generalizability experiments, a non-negativity threshold and normalization between 0 and 1 was applied to the reconstructed images. The synthetic dataset consisted of 3000 training images and 100 testing images from the MNIST and fashion-MNIST datasets.

In our experiments, we tested the unrolled network with and without the refinement U-Net and VGG-loss. When testing only the unrolled network, the batch size was equal to the training set size; however, when the U-Net and VGG-loss are applied, a smaller batch size is used to meet the greater memory demands and the GPU memory limitations. In addition, to meet the input size requirement of the U-Net and VGG-Net, the images were zero padded to increase the size from 41×41 to 64×64. The U-Net consisted of four downsampling levels (i.e. images were downsampled by a factor of $2^{4}=16$) followed by four upsampling levels to bring images back to size 64×64. Finally, the weight factor for the VGG perceptual loss was set to 0.01 for all experiments.

Computational processing used a combination of both CPU and GPU hardware. Highly parallelizable processes, including the model training, applying the learned forward model, and generating synthetic data with Monte Carlo, were performed on Nvidia 1080/2080 Ti’s. Other post-processing was performed on a standard desktop, running on an Intel Xeon CPU.

### 5.2 Real-World Data Capture Procedure

**Fig. 3:**
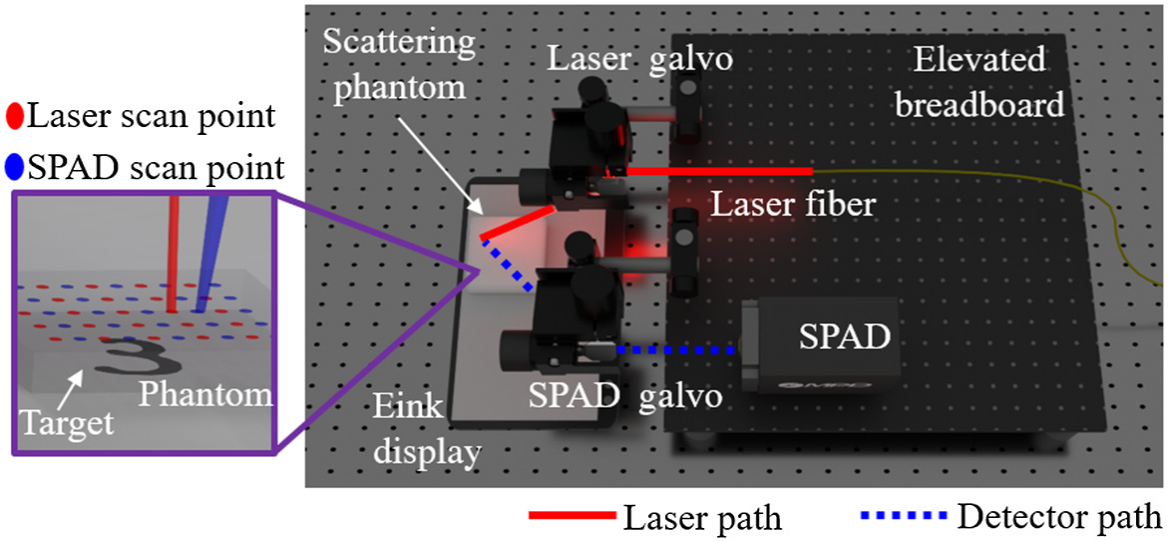
Unrolled-DOT experimental prototype setup: a rendering of our prototype system is shown above. Images displayed on an E-ink monitor served as the targets. A tissue phantom was placed in front of the target to mimic the effects of biological scattering. Our imaging setup consisted of a laser and a single pixel single photon avalanche diode (SPAD). The laser and virtual detector are raster-scanned over the surface of the tissue phantom by a pair of galvo mirrors.

Figure 3 shows a rendering of the prototype that was used to capture experimental data. Our setup consisted of a light source, detector, and target. For the light source, we used a pulsed laser (the NKT supercontinuum laser) set to 670 to 680 nm wavelength. Our detector is the MPD FastGatedSPAD, which is a single-pixel single-photon avalanche diode (SPAD). The detector also uses an associated MPD picosecond delayer and a Picoquant Hydraharp to perform the time-correlated single-photon counting.

Our target consisted of an E-ink display and an optically scattering tissue phantom. The E-ink display allows us to control the spatial distribution of light absorbers and scatterers programmatically.^74^ We used a Form 3 stereolithography three-dimensional (3D) printer to synthesize the optical tissue phantom. The optical properties can be controlled by mixing the Formlabs white and clear resins. By measuring the temporal point spread function of the transmitted light through the phantom, we determined the scattering coefficient of the tissue phantom to be $∼9\;{\mathrm{mm}}^{-1}$, which is within the acceptable range of biological tissue.^75^ More details regarding the phantom synthesis and characterization procedure can be found in our supplementary materials and the following references.^5^^,^^10^^,^^76^^,^^77^

We captured a total of 5000 measurements, which we split into a training and testing set of 4500 and 500 measurements, respectively. Ground truth targets consisted of examples from MATLAB’s digits dataset, which we then programmatically projected onto the E-ink display. The measurements consisted of 5×5 source and 5×5 detector scan points. The minimum source–detector separation was 4 mm, and the inter-source separation was 8 mm. In the raw data, each transient was initially binned into 4000 timebins, which were each 1-ps in duration. In post-processing, the data were further binned into 400 timebins, which were each 10 ps in duration. For all TD measurements, we applied temporal filtering, specifically the Laplace-temporal-filter^78^ with $s=10^{-4}$. This helped reduce the amount of data that needed to be processed and improved the image reconstruction quality. Because the temporal-filtering reduces the transient to a scalar value, the input is a B×N matrix, where B is the batch size and N is the number of source–detector pairs. For generating CW-DOT measurements, these time-domain transients can be integrated into a scalar. Using this system, we captured, to the best of our knowledge, the first dataset of DOT/ToF-DOT measurements with thousands of examples for training supervised machine learning models.

## 6 Results and Discussion

In this section, we report the results of our experiments, which include tests on both synthetic and real-world data. We began by testing the image reconstruction algorithm on a simulated dataset to show the benefits of applying a refinement U-Net and VGG-loss, and to show the algorithm’s generalizability. We also applied Unrolled-DOT to experimentally collected (real-world) data. Our first experiment on the real-world data is to verify the validity of our learned model itself. To do this, we visualize both the output feature maps of the pretrained VGG-loss as well as the learned model. In addition, we also demonstrate that our model is able to account for model mismatch by testing how the network performs when initialized with a misaligned source–detector array. After verifying our results, we conduct a performance analysis. The first test in this performance analysis is to compare the results of training the unrolled network and U-Net end-to-end versus training the U-Net after training the unrolled-network (two-step training). Next we conduct an ablation study, in which we test how the model performs when various training parameters are varied including the loss function, the number of training iterations, and number of layers. Finally, we compare our approach to traditional physics-based linear solvers as well as learning-based inverse solvers. We conducted this test on real-world data and compared both a standard ToF-DOT arrangement (measurements from all source–detector pairs) as well as a CToF-DOT.

### 6.1 Image Reconstruction on Synthetic Data

#### 6.1.1 Comparing performance with and without U-Net+VGG on simulated data

**Fig. 4:**
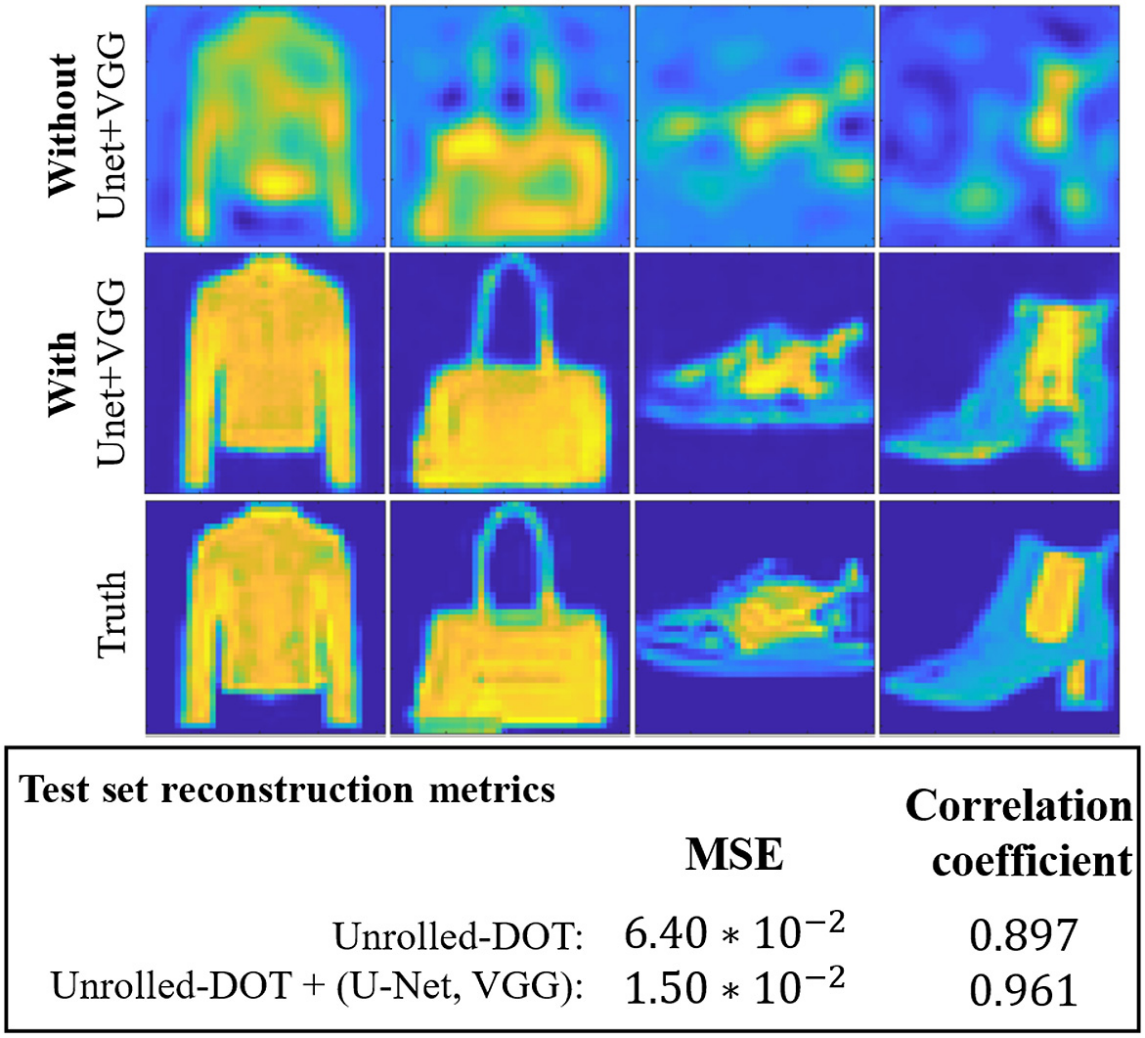
Improvement in image reconstruction quality using U-Net and VGG perceptual loss: We trained and tested our Unrolled-DOT algorithm on the fashion MNIST dataset. We compared the reconstruction quality with and without the refinement U-Net and VGG perceptual loss. We can see that incorporating the U-Net and VGG loss dramatically improves the reconstruction quality, based on both visual inspection and the average MSE across all test images. We have also shown that with these two enhancements, the reconstruction algorithm can accurately reconstruct images with varying levels of contrast.

We began by testing the performance of our model on synthetic data. We first demonstrated the benefits of adding a refinement U-Net and VGG perceptual loss to the unrolled network. Here, we trained solely on images from the fashion MNIST dataset. In both cases, a three-layer unrolled network was trained with a learning rate of $\mathrm{LR}=10^{-4}$ and MSE-loss. When training just the unrolled network a batch size equal to the training set (3000 measurements) was trained for 2000 iterations. With the U-Net and VGG-loss, the model was trained for 200 iterations with a batch size of 500 measurements. In Fig. 4, we see that adding the U-Net and VGG-loss to the network architecture dramatically improves the visual quality of the reconstructed image. The benefits of adding these components are further confirmed by quantitative metrics of the image reconstruction quality: the MSE and correlation factor. By incorporating the U-Net and VGG-loss, the MSE decreased from $6.40*10^{-2}$ to $1.50*10^{-2}$ while the correlation coefficient increased from 0.897 to 0.961. We have also shown that using Unrolled-DOT, it is possible to reconstruct more complex images that have varying contrast levels rather than sparse, binary images that are typically used as targets.

#### 6.1.2 Generalizability test

**Fig. 5:**
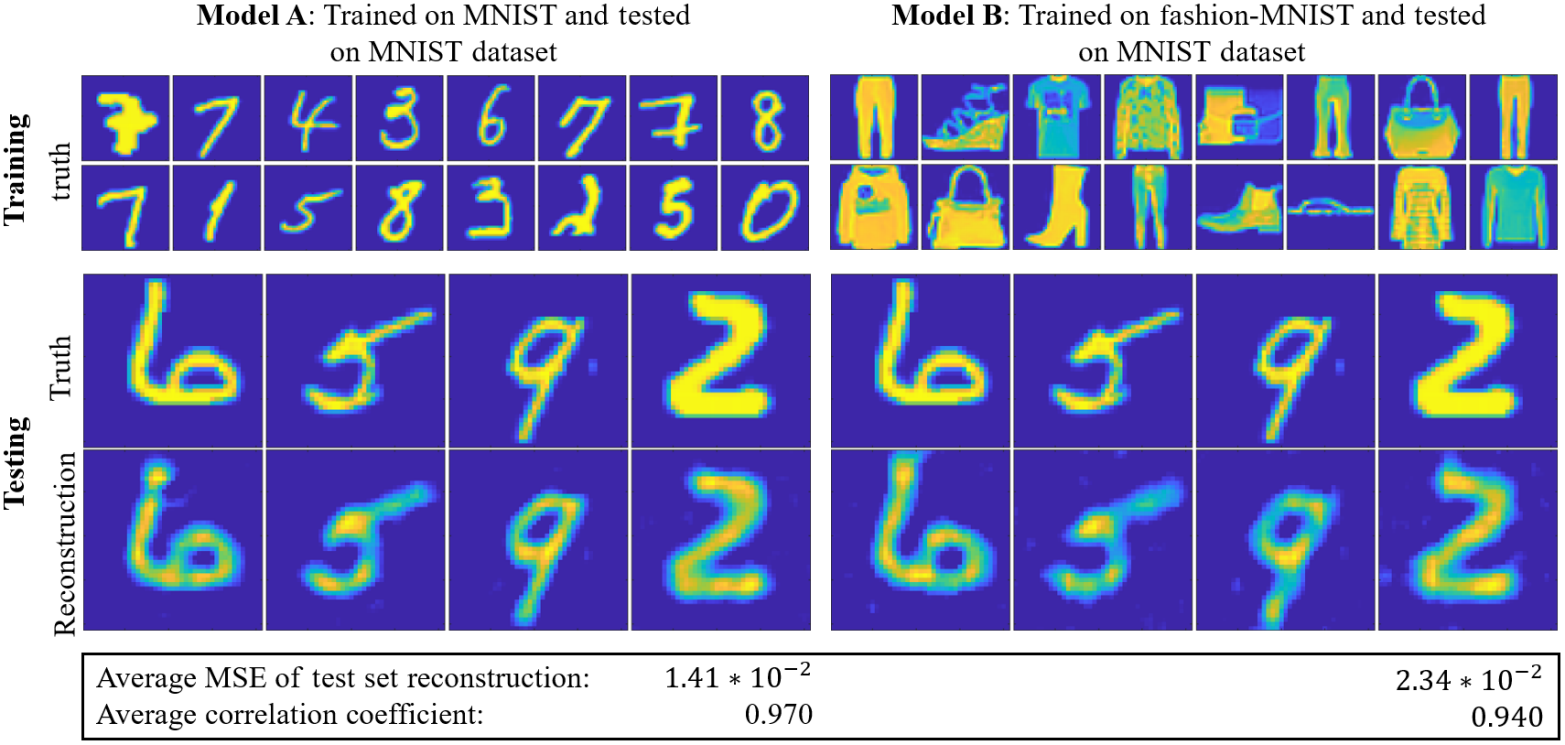
Generalizability of the Unrolled-DOT model. We trained two models: one on the MNIST dataset (model A) and another on the fashion MNIST dataset (model B). Both models performed similarity in reconstructing test images on the MNIST dataset, demonstrating the generalizability of our learning approach.

Next, we also demonstrated the generalizability of Unrolled-DOT. We trained two separate models on structurally different images: the MNIST and fashion-MNIST datasets, respectively. We then performed image reconstruction on measurements generated from MNIST dataset images. In this case, we tested only the unrolled-network without the U-Net and VGG-loss. We see that both models achieve good reconstruction of the test images, though model A does achieve slightly higher reconstruction quality than model B (an increase in the correlation coefficient from 0.94 to 0.97) because it is trained and tested on images of the same class (Fig. 5).

### 6.2 Interpretability of the Trained Model

#### 6.2.1 Visualization of the VGG-loss features

**Fig. 6:**
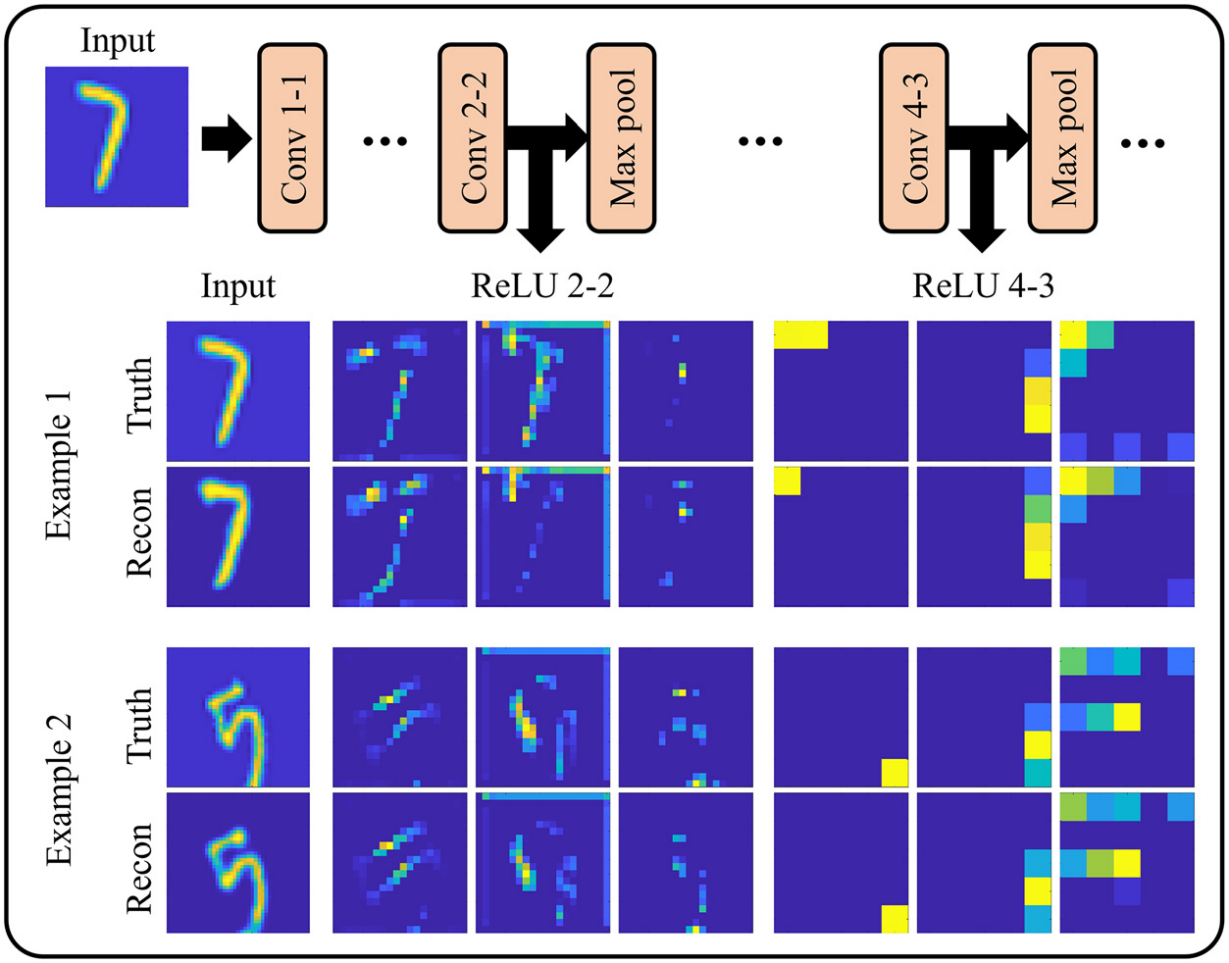
Visualization of VGG-network feature maps: The VGG loss uses the ReLU 2-2 (output of the ReLU between second convolution and second max pool) and ReLU 4-3 (output of the ReLU between fourth convolution and third max pool).^68^ Johnson et al.^72^ have noted that ReLU 2-2 corresponds to low-level features while ReLU 4-3 corresponds to higher-level structures. Thus, these two features were chosen for the VGG loss to encourage a better fit for both low-level features and overall structure.

In addition to a standard training loss, the Unrolled-DOT algorithm also uses a VGG-loss. To calculate the VGG-loss, the ground truth training images and reconstructed images are passed through a pretrained VGG network. The VGG-loss is then determined by the MSE/MAE error between the VGG network outputs for the ground truth and reconstructed images. Examples of feature maps that are generated by the VGG network are visualized in Fig. 6. The outputs used in the VGG loss are the ReLU 2-2 and the ReLU 4-3 features, which are the network outputs between the second convolution and second max pool and the fourth convolution and third maxpool, respectively.^68^^,^^72^ Johnson et al.^72^ observed that the ReLU 2-2 features corresponded to the low-level features of the image, while the ReLU 4-3 features appeared to correspond to the higher-level structures of the image. Therefore, using both features for the VGG-loss aids in preserving the fidelity of both low-level features and overall structure. We see in Fig. 6 that ReLU 2-2 seems to correspond to features of the input image, such as edges. While the ReLU 4-3 feature maps are very heavily downsampled, we see that for both sets of VGG outputs, there is good agreement between the ground truth and reconstructed images.

#### 6.2.2 Visualization of the learned Jacobian

The layers of our learned network correspond to the iterations of a standard linear solver. As a result, the learnable parameters can provide insight into the underlying physical parameters of the system. Recall from Sec. 4 that the learnable parameter W corresponds to the adjoint of the Jacobian matrix, i.e., $W=\gamma J^{T}$ in the unlearned algorithm. Therefore, by visualizing the learned W parameter, we can analyze the learned sensitivity matrix.

**Fig. 7:**
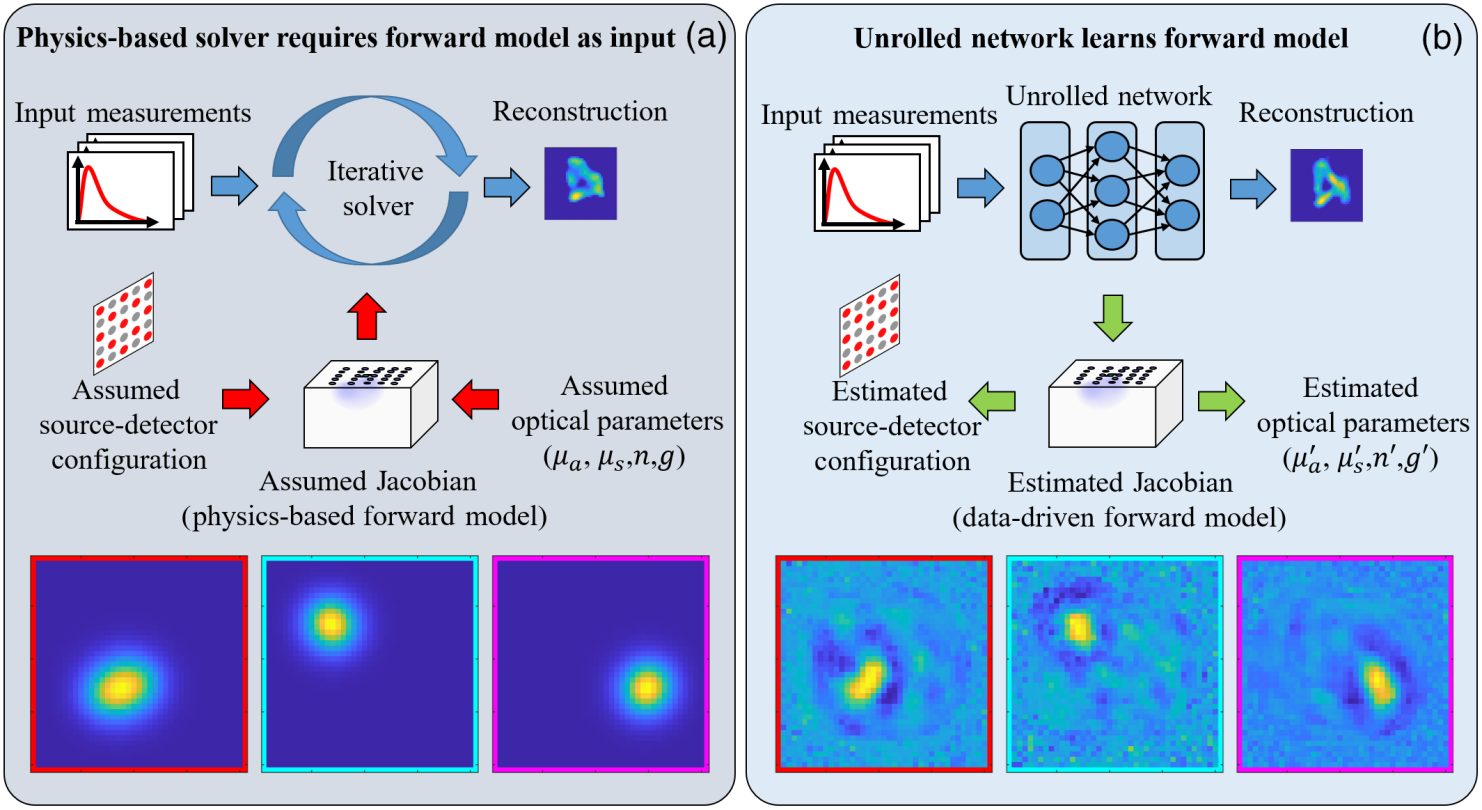
System parameter estimation using learned model. (a) In traditional DOT solvers, the forward model is assumed and must be passed in as an algorithm input. (b) With Unrolled-DOT, the forward model is estimated from data. Visualizations for rows of the physics-based and learned-forward models are shown on the bottom row.

In Fig. 7, we show a visualization of rows of the physics-based Jacobian matrix, and the learned sensitivity profile W, which was trained on real-world measurements. Because the Jacobian matrix describes the sensitivity of the measurements to small perturbations in the optical parameters, it can also be interpreted as the likelihood that a photon will pass through a particular point in space. In this visualization, we see the sensitivity profile as a function of (x,y) spatial location, for a fixed depth z. The regions of highest sensitivity, i.e., the bright yellow regions, correspond to the position of the light source/detector. This follows from the intuition that the sensitivity profile describes the likelihood of photons passing through a point in space: regions closer to the source/detector will have a higher likelihood of a photon passing through and therefore a greater sensitivity.

The interpretability of the learned model allows for greater insight into the underlying system. In future work, the learned Jacobian could be used to estimate parameters of the underlying physical model. This could be useful for calibrating the source–detector positions, or estimating the optical parameters.

#### 6.2.3 Mitigating model mismatch with Unrolled-DOT

**Fig. 8:**
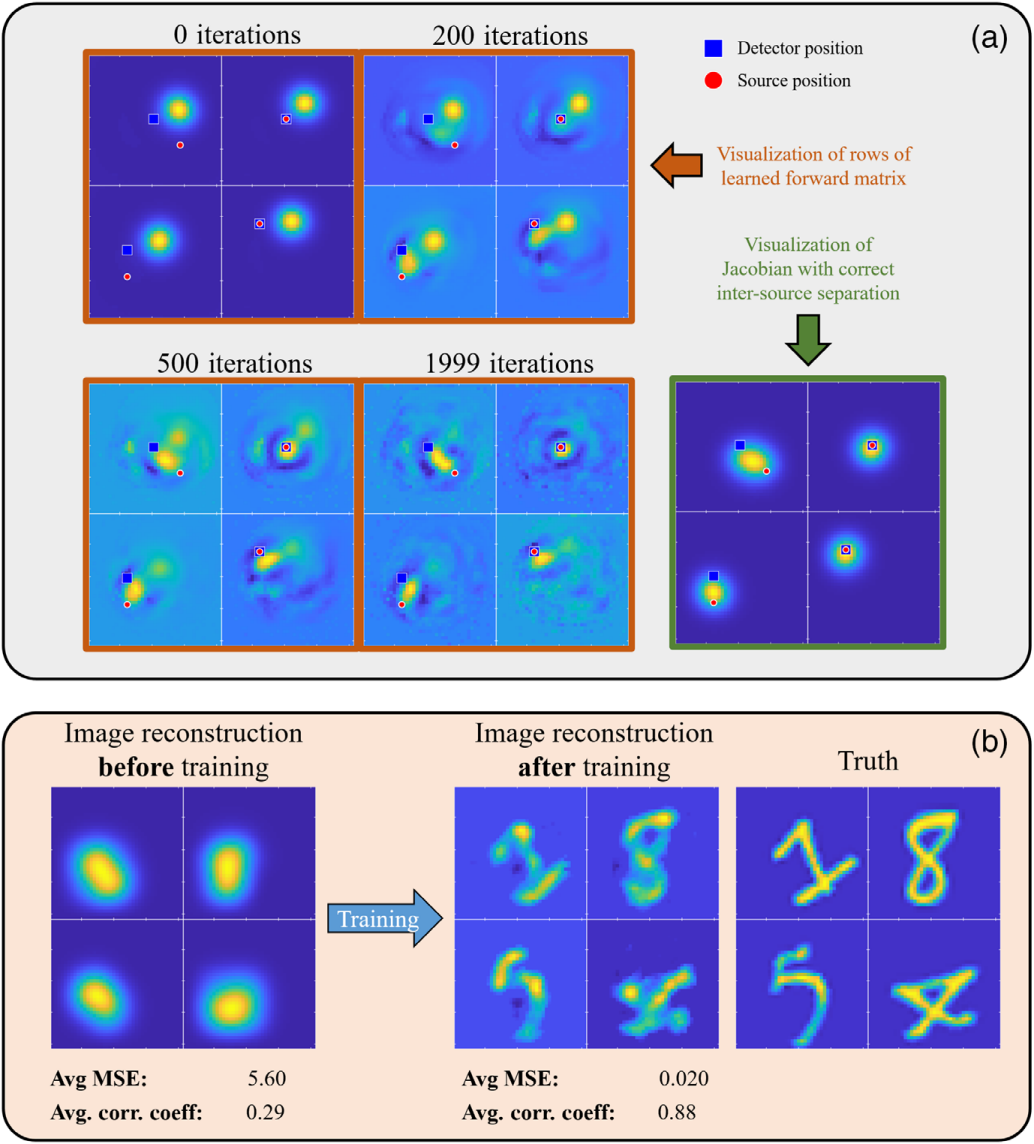
Overcoming model mismatch using Unrolled-DOT: the model was initialized with a weight matrix, which was generated with incorrect source–detector positions. Panel (a) shows a visualization of the rows of the forward matrix at different stages of training. The regions of highest sensitivity (bright yellow region) correspond approximately to the position of the source–detector pair. We see that at 0 iterations, the regions of highest sensitivity do not correspond well to the true source–detector positions. After 2000 iterations of training, we see there is good agreement between the source–detector positions and regions of high sensitivity. In addition, in panel (b), we see that training the network and reducing model mismatch also improves the image reconstruction quality.

We also wish to demonstrate that Unrolled-DOT is able to mitigate certain cases of model mismatch through training. To demonstrate this, we use an incorrect forward matrix to initialize the Unrolled-DOT model, as shown in Fig. 8. In Fig. 8(a), we visualize the layers of the unrolled network. More specifically, we visualize rows of the matrix, which correspond to the network layer. Recall that the layers of the Unrolled-DOT model correspond to the Jacobian, or sensitivity matrix. The rows of this matrix should correspond to the sensitivity for a specific source–detector pair. Therefore, we should expect to see highest sensitivity (bright yellow regions) at locations corresponding to the source–detector position. The true positions of the sources and detectors are overlaid on the visualization of the network layers as a red circle and blue square, respectively. Initially, we see that the regions of high sensitivity do not correlate well with the source–detector positions. However, after 2000 iterations of training, we see that the regions of highest sensitivity shift to the correct locations, corresponding to the source–detector positions. As expected, in Fig. 8(b), we see that mitigating this model mismatch also improves the image reconstruction quality. The average MSE is reduced from 5.6 to 0.020 and the average correlation coefficient is increased from 0.29 to 0.88.

### 6.3 Performance Analysis

#### 6.3.1 End-to-end training versus two-step training

**Fig. 9:**
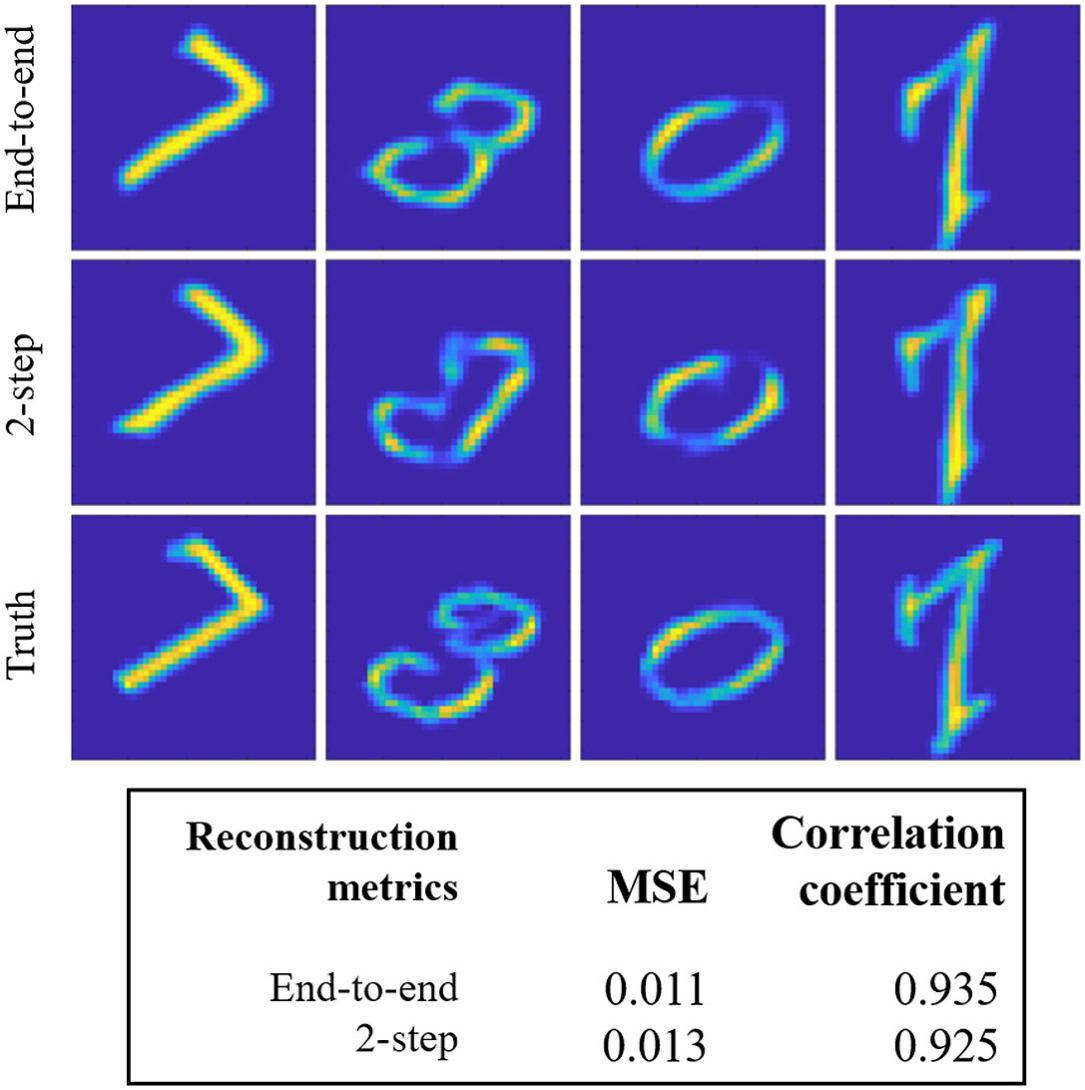
Comparison of end-to-end training with two-step training procedure. The Unrolled-DOT algorithm uses two networks: an unrolled network and a U-Net. The two networks can be trained end-to-end or in two steps: training the unrolled network first, then the U-Net. Above, average reconstruction MSE and correlation coefficients are reported for the full test set. We see there is only a marginal reduction in the MSE from 0.013 with the two-step training to 0.011 with the end-to-end training and a 0.01 increase in the correlation coefficient. Visual inspection of the example reconstruction images (shown above) appears to confirm that both methods achieve similar reconstruction quality.

The Unrolled-DOT model consists of two networks: an unrolled network and a U-Net. These two networks can be trained end-to-end or in a two-step procedure, training the unrolled network first and subsequently the U-Net. The first test in our performance analysis is to compare the end-to-end training procedure with the two-step training procedure. The results of this experiment are shown in Fig. 9. The end-to-end training procedure achieves a small improvement compared to the two-step training procedure; however, the improvement is relatively small since the MSE is only reduced from 0.013 to 0.11. From the reconstructed images shown in Fig. 9, we also see that the two methods achieve comparable image reconstruction quality.

#### 6.3.2 Ablation study

In addition to performing image reconstruction, we also characterized the performance of our algorithm as a function of different training parameters. Our ablation study is conducted on real-world data. For simplicity, we tested only the unrolled network, without the U-Net or VGG-loss. In general, we trained a three-layer unrolled network for 2000 iterations with MAE loss, and a learning rate of $\mathrm{LR}=2*10^{-4}$. The parameter is varied if it is the dependent variable for a particular experiment. For example, in testing the impact of the number of network layers, the model is still trained for 2000 iterations with MAE loss, and a learning rate of $\mathrm{LR}=2*10^{-4}$; however, the number of layers is varied between 1 and 10.

**Fig. 10:**
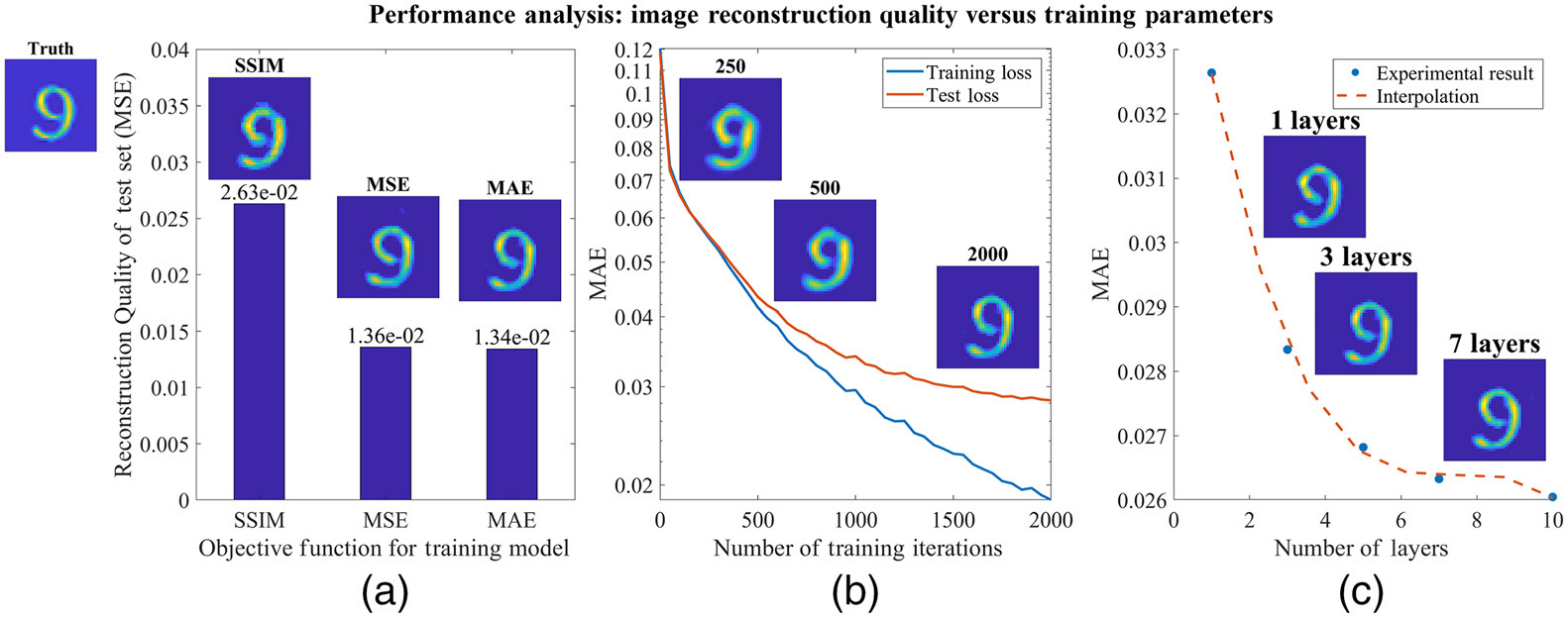
Ablation study on objective function, number of training iterations, and number of network layers: we tested the image reconstruction quality as a function of several training parameters. (a) The MAE loss provides marginal improvements in the image reconstruction quality compared to SSIM and MSE. (b) In addition, with a reasonable learning rate, the reconstruction quality increases with more iterations in training. (c) Finally, we found the image reconstruction quality increases with the number of layers, though there seem to be diminishing returns beyond seven layers.

In this analysis, we specifically studied the performance as we varied the loss function, number of training iterations, and the number of layers in our network (Fig. 10). First, we compared three different loss functions. The loss functions we chose were based on the standard metrics used to judge image reconstruction quality: the MSE, the mean absolute error (MAE), and the SSIM loss.^79^^,^^80^ In comparing the loss functions, it appears the MAE produces the highest quality reconstructions, with less blurring and artifacts as compared to MSE and SSIM. It is interesting that the MAE ($L^{1}$) objective achieves a lower overall MSE on the test set than the MSE ($L^{2}$) objective; however, this phenomenon was also observed by Zhao et al.^81^ They postulated this is because MAE is able to avoid local minima better than MSE.

Second, we tested the number of iterations used to train the network. From this experiment, we found a fairly straightforward result: given an appropriate learning rate, the image reconstruction quality increased as the number of iterations increased. In Fig. 10, this is particularly noticeable in the reconstruction of the number “9,” in which we see that the blurring effect is significantly decreased as the number of iterations is increased.

Third, we tested how the number of layers in our learned model affects the reconstruction quality. We found that by increasing the number of layers, there is an increase in the image reconstruction quality, which is in agreement with the standard intuition and prior work.^16^ However, this improvement does seem to produce diminishing returns as the number of layers is increased beyond seven layers.

### 6.4 Image Reconstruction on Experimentally Collected Data

We trained and tested our model on real-world data that we collected from our prototype system. The model was trained on a dataset of handwritten digits. Our model was tested on measurements from both a standard ToF-DOT setup (i.e., scanning all pairs of sources and detectors), as well as a CToF-DOT. Our Unrolled-DOT model was trained with a learning rate $\mathrm{LR}=2*10^{-4}$. Measurements are normalized to 5.0. With the U-Net and VGG-loss, the model was trained for 400 iterations with a batch size of 900 measurements. For training just the unrolled network, the model was trained for 2000 iterations with a batch size equal to the training set size (4500 measurements).

To show the benefits of our approach, we compared our method with two physics-based inverse solvers and two machine-learning-based inverse solvers. The physics-based solvers that we compare against are FISTA with $L^{1}$ sparsity for CToF-DOT reconstruction, which was proposed by Zhao et al.,^5^ and ADMM with total-variation (TV) regularization for DOT reconstruction, which was proposed by Lu et al.^82^ We used in-house implementations for each of these inverse solvers and applied the Monte Carlo forward model. For FISTA, both the Jacobian and measurements are scaled to 1.0; while for ADMM the Jacobian and measurements are normalized to $2.1*10^{-4}$ and $6*10^{5}$, respectively. Since the solvers are applied to a linear inverse problem, the normalization factors should not impact the final reconstruction as long as the non-linear hyperparameter terms are scaled appropriately. Additional hyperparameters for the physics-based solvers are listed in the supplementary material.

In addition, we also compare our method with two machine-learning-based methods: a standard FC multilayer perceptron, which will be referred to as the FC-net, and the AUTOMAP architecture.^45^ FC-net possesses an input layer, output layer, and a single hidden layer of width equal to the output size and uses the tanh activation function. FC-net is trained with MSE-loss. We also compare our method with AUTOMAP. Similar to FC-net, the first portion of the AUTOMAP network consists of FC layers: an input layer, and two hidden layers with width equal to the output size, and tanh activation functions. After the FC layers, the intermediate vector is reshaped into a two-dimensional image with dimensions equal to the output. The network then applies two convolutional layers with a kernel size of 5 and ReLU activation functions, followed by a deconvolution layer with a kernel size of 7. AUTOMAP is also trained with MSE-loss. More details about the AUTOMAP architecture are provided by Zhu et al.^45^

#### 6.4.1 Unrolled-DOT for confocal scanning geometry

**Fig. 11:**
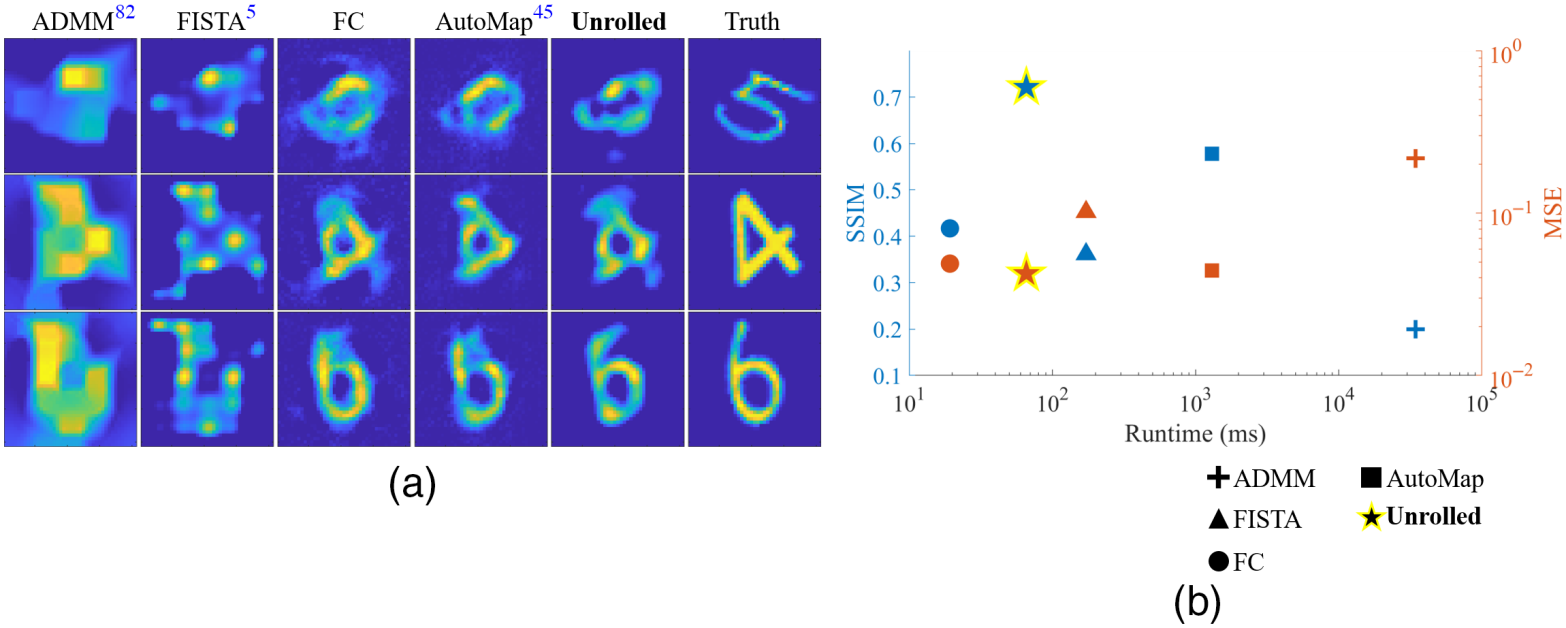
Unrolled-DOT image reconstruction on experimental data with sparse 5×5 confocal scanning geometry: the reported metrics are averaged over the entire test set. In panel (b), we see there is a dramatic reduction in the MSE from 0.102 using FISTA and 0.218 with ADMM, compared to 0.042 with our approach. Similarly the SSIM increases from 0.20 and 0.36 for ADMM and FISTA, respectively to 0.72 with our approach. In addition, Unrolled-DOT achieves a faster runtime than AUTOMAP and an increased SSIM and reduced MSE compared to both AUTOMAP and the FC network. Visually, in panel (a), we also see from the reconstructed images that the Unrolled-DOT algorithm shows good agreement with the ground truth and removes artifacts from the sparse source–detector array.

We first tested Unrolled-DOT on a sparse set of measurements, collected from a 5×5 array of sources and detectors with a CToF-DOT. Therefore, measurements from only 25 source–detector pairs are used for image reconstructions. For this experiment, we used a three-layer unrolled network without the U-Net and VGG-loss and trained with MSE-loss. The results are shown in Fig. 11. From the reconstructed images in Fig. 11(a), we see that Unrolled-DOT achieves a higher image reconstruction quality compared with all other methods. This is particularly true compared to the physics-based solvers, which display severe reconstruction artifacts. In Fig. 11(b), we show a plot of the reconstruction quality metrics and reconstruction runtime. Each algorithm corresponds to a different symbol, shown on the key in the bottom right. The left y-axis (blue) corresponds to SSIM, while the right y-axis (red) corresponds to MSE. A higher SSIM and lower MSE correspond to improved image reconstruction quality, while lower runtime corresponds to a faster algorithm. Therefore, the “ideal” algorithm would be in the top left and bottom left for the blue and red plots, respectively. Based on the increase in the SSIM and the reduction in the MSE, shown in Fig. 11(b), we see that Unrolled-DOT outperforms all other algorithms in this comparison (exact SSIM, MSE, and runtime values are included in our supplementary material). Unrolled-DOT is also faster than all other algorithms except the FC-net; however, the increased speed of FC-net comes at the cost of reduced image reconstruction quality compared to Unrolled-DOT.

#### 6.4.2 Unrolled-DOT for reconstruction of full ToF-DOT measurements

**Fig. 12:**
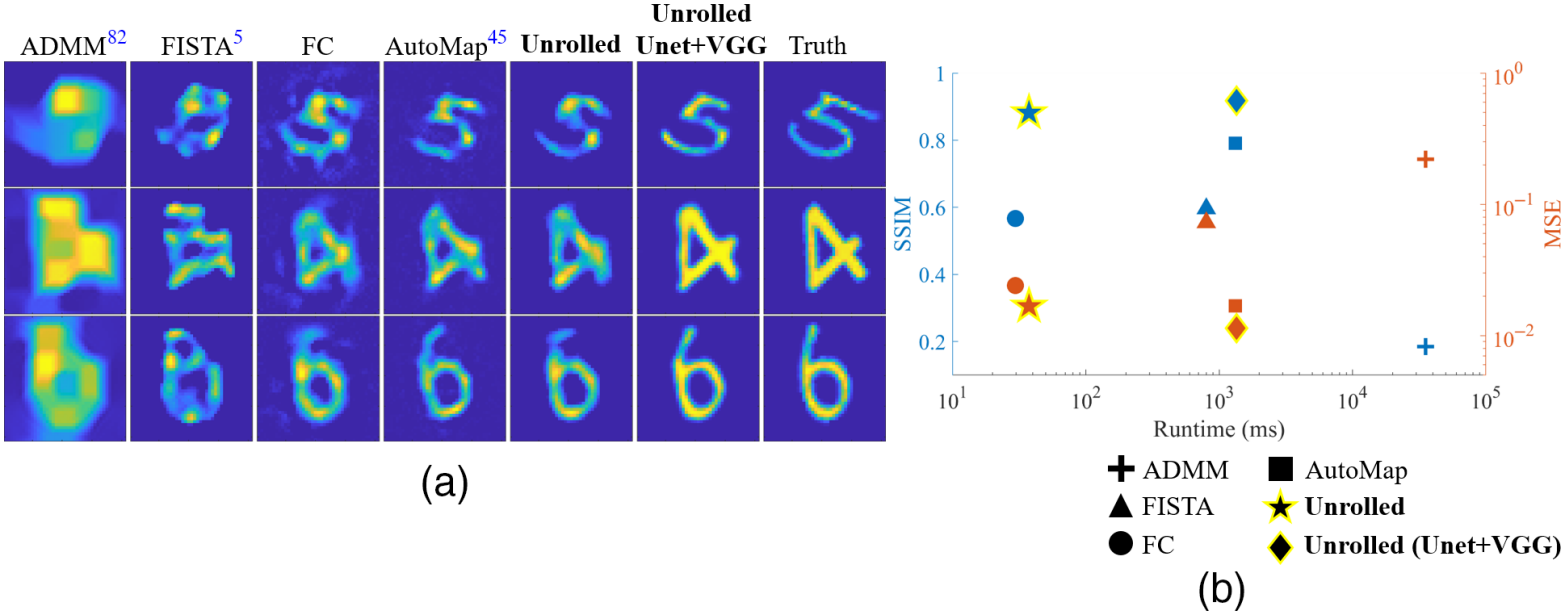
Unrolled-DOT image reconstruction on experimental data from 625 source–detector pairs. We demonstrate the improvements both visually (a) and in the reconstruction metrics and runtime (b). Unrolled-DOT outperforms traditional physics-based linear solvers since it avoids imaging artifacts and poor reconstruction quality that results from model mismatch and sub-optimal hyperparameters. In addition, we see that the unrolled network, even without the U-Net and VGG loss, achieves better reconstruction quality than the fully connected network and comparable image reconstruction quality to AUTOMAP, but with significantly reduced runtime. With the U-Net and VGG loss, Unrolled-Dot achieves a significantly higher SSIM and lower MSE.

Next, we tested the Unrolled-DOT algorithm on a full set of ToF-DOT measurements, i.e., all source–detector pairs. The measurements were captured from a 5×5 source array and 5×5 detector array. Therefore, measurements were collected from 25 sources and 25 detectors, a total of 625 source–detector pairs. Here, we tested a 1-layer unrolled network with MAE loss. We tested the algorithm with and without the U-Net and VGG-loss. From the reconstructed images in Fig. 12, we see that our Unrolled-DOT method achieves a noticeably improved image reconstruction. In addition to visual inspection, we also see the performance of our method using quantitative metrics: the SSIM and MSE [shown in Fig. 12(b) and Table S2 in the Supplementary Material]. We see that, even with just a one-layer unrolled network, the SSIM increases from 0.18 to 0.60 with ADMM and FISTA, respectively, to 0.88 with Unrolled-DOT. In addition, when the U-Net and VGG-loss are added to our training pipeline, the image reconstruction quality further increases.

There are two primary reasons for our improved reconstruction quality. First, our trained method is able to learn the underlying physical model and can avoid potential model mismatch. Various parameters of the imaging system and target are automatically calibrated. This includes the source–detector positions and optical parameters of the target. Secondly, our trained model directly optimizes the hyperparameters for image reconstruction. In contrast, traditional linear solvers require several hand-tuned parameters as inputs. A full table of all relevant hyperparameters and the procedure for tuning these parameters is included in the supplementary materials, Table S1 in the Supplementary Material. At a minimum, this includes tuning the number of iterations and the weight of the regularization terms. For the FISTA algorithm, we incorporated $L^{1}$ regularization to tune the image sparsity; for the ADMM algorithm, we incorporated TV-regularization. Since these hyperparameter values are generally hand-tuned, the performance of the corresponding inverse solver will depend on the tuning heuristics, which may not be optimal. As a result, the performance is improved using a learning-based algorithm that directly optimizes the hyperparameters.

In Fig. 12(b). we also report the runtimes to reconstruct the full test set, which consists of 500 images. This plot follows a similar format to Fig. 11(b). We see that the unrolled network is over an order of magnitude faster than other physics-based linear solvers. This speed-up was achieved with all algorithms in the comparison running on the same hardware: the Intel Xeon CPU. We will note that there were also implementation-specific details across the different methods. First, the learning-based methods were implemented with Python and Pytorch, while the other methods used MATLAB. In addition, the TV-regularization for the ADMM reconstruction used a computationally expensive matrix multiplication to calculate the image gradient. However, the speed-up from using our approach is not only demonstrated by the timing experiments. Unrolled-DOT requires just one layer to obtain the desired image reconstruction. FISTA and ADMM may require tens of iterations. Because Unrolled-DOT can achieve high-quality reconstruction with just one layer, and each layer performs a comparable number of operations to a single iteration of a standard solver, our method should be faster regardless of implementation-specific variations.

We also see that the refinement U-Net and VGG-perceptual loss further improve the image reconstruction quality. With these enhancements, the model seems to be better at rejecting spurious artifacts and increasing the sharpness and contrast. Quantitatively, we see in Table S2 in the Supplementary Material that this led to an increase in the SSIM from 0.88 to 0.92 and a decrease in the MSE from $1.67*10^{-2}$ to $1.14*10^{-2}$. Though the U-Net does slow down the algorithm, it is useful in applications that prioritize the reconstruction quality over the run time. Furthermore, the benefits of the U-Net and VGG-loss should be more distinct for image reconstruction on more complex natural images.

Finally, we see that our model performs well compared to other trained models. Without the U-Net and VGG-loss, Unrolled-DOT is faster than all other algorithms in our comparison, except the FC-Net. However, it is able to achieve an increased SSIM and reduced MSE compared to the FC-Net and comparable image reconstruction quality to AUTOMAP. With the addition of the U-Net and VGG-loss, the reconstruction metrics are further improved compared to AUTOMAP and FC-Net.

## 7 Conclusion

We have proposed a learning-based method for solving the DOT inverse problem. Our method is able to obtain high-quality image reconstructions through densely scattering media, perform reconstructions efficiently, and can be trained end-to-end on learned data. Additionally, our network is interpretable, which may aid in system calibration. We demonstrated our results on both synthetic and real-world data, and prepared a dataset of 5000 ToF-DOT measurements. Ultimately, our learned model is a step towards fast high-resolution imaging through scattering media.

## Code, Data, and Materials Availability

Code is available at: https://github.com/yyiz/Unrolled-DOT. Our real-world DOT/ToF-DOT dataset is available at the following link: https://doi.org/10.5281/zenodo.7654959 (Ref. 19).
