# Supplementary material for: Unrolled-DOT: an interpretable deep network for diffuse optical tomography
Source: J Biomed Opt. 2023 Mar 8;28(3):036002. doi: 10.1117/1.JBO.28.3.036002 (PMC9995139; doi:10.1117/1.JBO.28.3.036002)
Supplement: Supplementary file 1 [file JBO_028_036002_SD001.pdf]

Unrolled-DOT: An Interpretable Deep Network for Diffuse Optical Tomography – Supplementary Material

Yongyi Zhao[a,*], Ankit Raghuram[a], Fay Wang[b], Stephen Hyunkeol Kim[c,d], Andreas Hielscher[d], Jacob T. Robinson[a], Ashok Veeraraghavan[a]
[a]Department of Electrical and Computer Engineering, Rice University, 6100 Main Street MS 366, Houston TX, 77005
[b]Department of Biomedical Engineering, Columbia University, New York, NY 11027
[c]Department of Radiology, Columbia University Irvine Medical Center, New York, NY 10032
[d]Department of Biomedical Engineering, New York University – Tandon School of Engineering, New York, NY 10010

1 Tissue Phantom Characterization

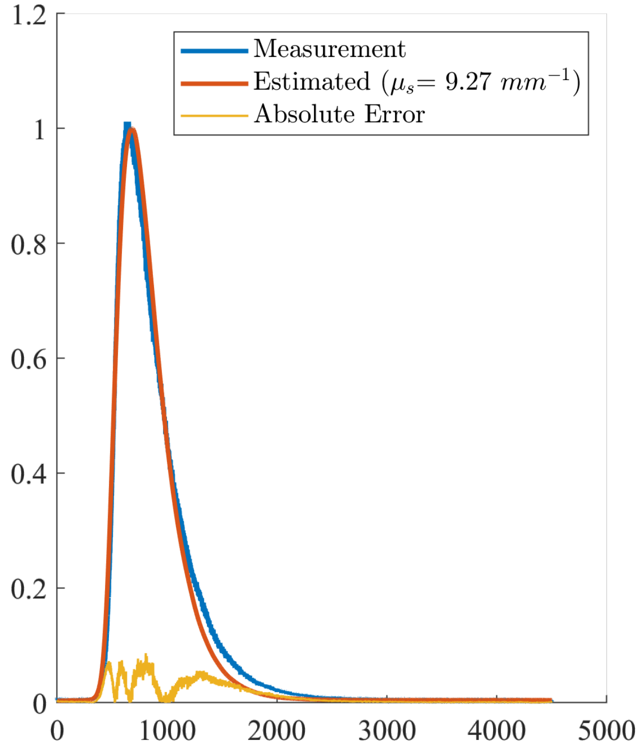

Fig S1 Tissue phantom characterization: The experimentally measured temporal point spread function (TPSF) is shown in blue, while the predicted TPSF is shown in red. We see that the experimental TPSF shows good agreement with the TPSF corresponding to a scattering coefficient, $\mu_s = 9.27mm^{-1}$ (error is plotted in orange).

Our tissue phantoms are synthesized by mixing the clear and white Formlabs resins, which allows us to tune the scattering coefficient. We then 3D print and characterize the solid tissue phantom. To characterize our phantom, we adapt the time-of-flight-based procedure originally described by Bouchard et al.,[76] and subsequently adapted in later works.[5,10] The premise of this approach is to use the broadening of the temporal point spread function (TPSF) as light undergoes increasing amounts of scattering. We first record a TPSF through a tissue phantom in transmission mode. The recorded TPSF is then fit to a simulated TPSF that is generated by a Monte Carlo

simulation for the same geometry. Since increasing the scattering coefficient (μ_s) increases the TPSF broadening, the predicted μ_s corresponds to the simulated TPSF that best fits the measured TPSF.

To achieve a good fit with the simulated TPSF, we should also match the tissue phantom geometry, the absorption coefficient (μ_a), and the refractive index (n). In this experiment, we 3D printed a tissue phantom in a slab geometry with dimensions $3cm \times 3cm \times 2cm$ and recorded the TPSF through $2cm$ of the tissue phantom. We used the values $\mu_a = 0.01mm^{-1}$ and $n = 1.46$ based on the experimental characterization of the Formlabs resin by Dempsey et al.[77] Finally, similar to Bouchard et al., we used the Levenberg-Marquardt algorithm to fit the experimental and simulated TPSF.[76] Our tissue phantom characterization result is shown in figure S1. For an absorption coefficient $\mu_a = 0.01mm^{-1}$ and refractive index $n = 1.46$, we determined the scattering coefficient $\mu_s \approx 9.27mm^{-1}$.

2 Image reconstruction on circular phantoms

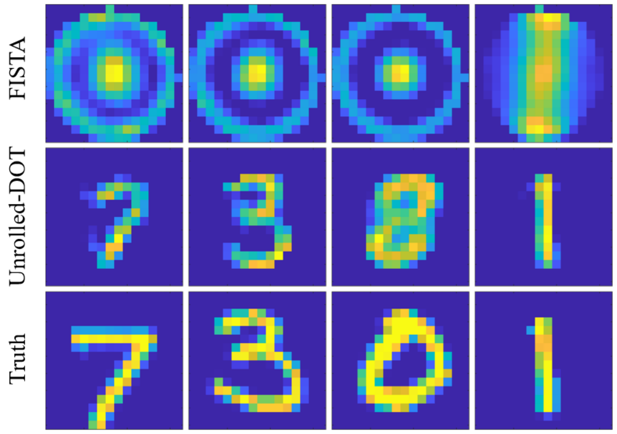

Reconstruction Metrics	MSE	Correlation Coefficient
FISTA	0.274	0.180
Unrolled-DOT	0.089	0.705

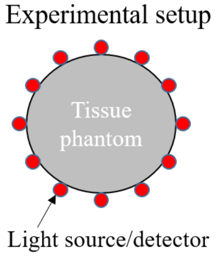

Fig S2 Unrolled-DOT image reconstruction on circular phantom: While most of the results presented thus far were for a slab tissue phantom in reflection mode, here we show reconstructions for a circular tissue phantom in transmission mode. From the example reconstructed images, we see that our model achieves a higher fidelity to the ground truth compared to FISTA. In addition, our method achieves a reduction in the MSE from 0.274 with FISTA to 0.089 with our model, and an increase in the correlation coefficient from 0.180 to 0.705.

While most of our experiments were conducted for a slab phantom with reflection mode geometry, we also wanted to test different tissue phantom configurations. In simulation, we also tested a circular phantom. As mentioned in the main document, 32 source-detector pairs are distributed over the surface of the tissue phantom. The measurements are not collected from all

source-detector pairs, only the source-detector pair that are placed on opposite sides of the tissue phantom. The imaging targets are obtained from the MNIST dataset, downsampled to 16×16, and placed within a circular tissue phantom.

Fig. S2 shows the results of our experiment. Here, we compare the results with the FISTA inverse solver. From the image reconstructions that are shown, Unrolled-DOT clearly outperforms FISTA in image reconstruction quality. The FISTA image reconstructions possess circular artifacts, which are likely due to the phantom geometry and source-detector placement. Based on the metrics, we also see a clear improvement in the Unrolled-DOT image reconstructions: a reduction in MSE from 0.274 to 0.089 and an increase in the correlation coefficient from 0.18 to 0.705.

3 Testing relationship between number of layers and image reconstruction quality

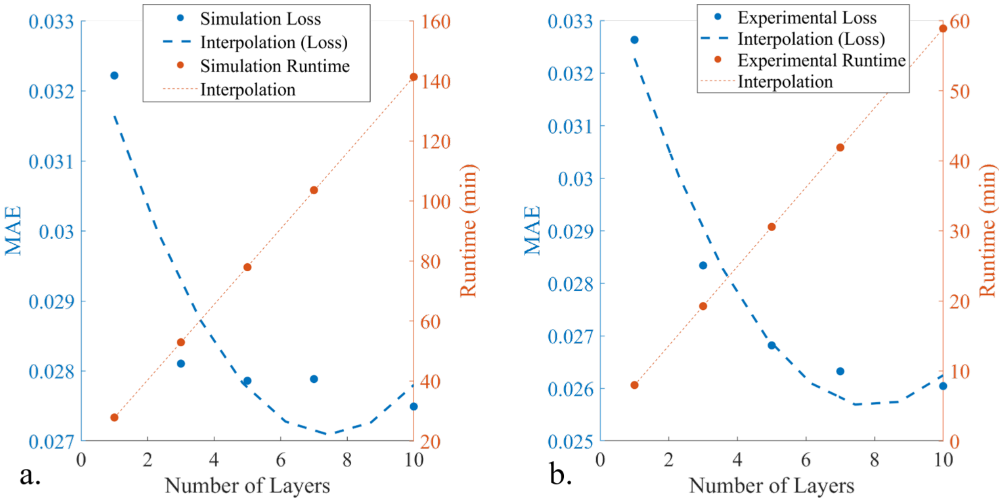

Fig S3 Benefits and limitations of increasing the number of network layers: The diminishing returns in image reconstruction quality and linear increase in the reconstruction time with more layers suggest that a network with greater than 5 layers would not be desirable. Therefore, using fewer network layers (just 3-5 layers) may be beneficial since the small reduction in error is likely offset by the increase in runtime.

We tested the image reconstruction quality as a function of the number of layers in the unrolled network. Here, to demonstrate a setup similar to the experimental result, we compared reconstruction with measurements from a 5×5 source-detector array. The model was trained on $10,000$ examples and tested on 500 examples with a learning rate of $1.8 * 10^{-4}$ and measurement normalization of 5.0.

In Fig. S3, we compare the image reconstruction quality versus the number of network layers in the model. We perform this comparison for both simulated (Fig. S3a.) and experimental (Fig. S3b.) data. The experimental data was obtained from the ablation study in section 6.3. For both models, we see there is generally a decrease in the error as the number of layers increases. For simulated data, the MAE decreases from 0.0322 at 1 layer to 0.0275 at 10 layers and for the experimental data it decreases from 0.0326 at 1 layer to 0.0260 at 10 layers. However, there appears to be diminishing returns beyond 5 layers and 7 layers for the simulated and experimental data, respectively. In addition, the algorithm runtime increases linearly with the number of layers.

Reconstruction Algorithm	Hyperparameter	Parameter Value
ADMM	Max Iterations	70
	τ_{incr}	1.2
	τ_{decr}	1.2
	μ	0.8
	ρ_{0+} (penalty term for non-negativity)	0.5
	Lagrange multiplier initial value - Non-negativity	$1 * 10^{-2}$
	ρ_{TV} (penalty term for total-variation)	$1 * 10^{-2}$
	Weight term for total-variation	5.0
	Lagrange multiplier initial value - total-variation	$1 * 10^{-2}$
	ρ_{TV} (penalty term for L1-norm)	$5 * 10^{-2}$
	Weight term for L1-norm	0.0
	Lagrange multiplier initial value - L1-norm	$1 * 10^{-2}$
FISTA (All source-detector pairs)	Max Iterations	30
	L1-penalty weight	1.0
FISTA (Confocal)	Max Iterations	20
	L1-penalty weight	$5 * 10^{-2}$

Table S1 Hyperparameter values: Above, we provide a table of the most relevant hyperparameters for the linear inverse solvers. The variable names listed in the ADMM section correspond to the notation used by Boyd et al.[67]

Therefore, particularly for applications in which high temporal resolution is critical, a network with 3-5 layers may be appropriate.

4 Linear Solver Hyperparameters

Table S1 shows the set of relevant hyperparameters for the untrained inverse solvers in our comparisons test. Since the parameters correspond to physically-meaningful aspects of the image, they can be tuned by hand. For example, the L1-norm penalty increases image sparsity. Therefore increasing the corresponding weight can aid in denoising and removal of spurious artifacts. Because hand-selecting parameters may not guarantee optimality, a training procedure that directly optimizes the hyperparameters may improve the image reconstruction quality.

5 Image Reconstruction on Real-World Data: Quantitative Metrics

Table S2 shows the corresponding metrics for the image reconstruction on real-world data from our test dataset. The algorithm runtimes correspond to the full runtime for reconstructing 500 images. The reported SSIM and MSE metrics correspond to the average metrics across all 500 images.

6 Supplementary Information on ToF-DOT/DOT Dataset

Our dataset is available at.[19] The dataset consists of 5000 total measurements. For each measurement, a time-of-flight transient was collected at 5×5 source positions and 5×5 detector positions, resulting in $5^4 = 625$ source-detector pairs. After post-processing, each transient consists of 400 timebins with a 10 ps binwidth. This results in an overall data array of size $400 \times 625 \times 5000$

Reconstruction Algorithm	SSIM	MSE	Runtime (ms)
Confocal Geometry			
ADMM	0.20	$2.18 * 10^{-1}$	$3.45 * 10^{4}$
FISTA	0.36	$1.02 * 10^{-1}$	$1.72 * 10^{2}$
FC	0.42	$4.89 * 10^{-2}$	$1.92 * 10^{1}$
Automap	0.58	$4.42 * 10^{-2}$	$1.30 * 10^{3}$
Unrolled-DOT	0.72	$4.24 * 10^{-2}$	$6.58 * 10^{1}$
All Pairs sources and Detectors			
ADMM	0.18	$2.21 * 10^{-1}$	$3.52 * 10^{4}$
FISTA	0.60	$7.26 * 10^{-2}$	$8.00 * 10^{2}$
FC	0.57	$2.41 * 10^{-2}$	$2.96 * 10^{1}$
Automap	0.79	$1.69 * 10^{-2}$	$1.32 * 10^{3}$
Unrolled-DOT	0.88	$1.67 * 10^{-2}$	$3.75 * 10^{1}$
Unrolled-DOT (U-Net+VGG)	0.92	$1.14 * 10^{-2}$	$1.35 * 10^{3}$

Table S2 Quantitative metrics for Unrolled-DOT image reconstruction on experimental data: Above, we show a table of the image reconstruction quality (measured by the SSIM and MSE) and the algorithm runtime. The values shown here correspond to image reconstructions on real-world test data shown in our main manuscript.

corresponding to the number of timebins, number of source-detector pairs, and number of measurements, respectively. Each measurement also consists of two data arrays for the background and perturbed state.
